# Influenza A virus polymerase acidic protein E23G/K substitutions weaken key baloxavir drug-binding contacts with minimal impact on replication and transmission

**DOI:** 10.1371/journal.ppat.1010698

**Published:** 2022-07-13

**Authors:** Jeremy C. Jones, Bogdan Zagribelnyy, Philippe Noriel Q. Pascua, Dmitry S. Bezrukov, Subrata Barman, Faten Okda, Richard J. Webby, Yan A. Ivanenkov, Elena A. Govorkova

**Affiliations:** 1 Department of Infectious Diseases, St. Jude Children’s Research Hospital, Memphis, Tennessee, United States of America; 2 Department of Chemistry, Lomonosov State University, Moscow, Russian Federation; University of Maryland, UNITED STATES

## Abstract

Baloxavir marboxil (BXM) is approved for treating uncomplicated influenza. The active metabolite baloxavir acid (BXA) inhibits cap-dependent endonuclease activity of the influenza virus polymerase acidic protein (PA), which is necessary for viral transcription. Treatment-emergent E23G or E23K (E23G/K) PA substitutions have been implicated in reduced BXA susceptibility, but their effect on virus fitness and transmissibility, their synergism with other BXA resistance markers, and the mechanisms of resistance have been insufficiently studied. Accordingly, we generated point mutants of circulating seasonal influenza A(H1N1)pdm09 and A(H3N2) viruses carrying E23G/K substitutions. Both substitutions caused 2- to 13-fold increases in the BXA EC_50_. EC_50_s were higher with E23K than with E23G and increased dramatically (138- to 446-fold) when these substitutions were combined with PA I38T, the dominant BXA resistance marker. E23G/K-substituted viruses exhibited slightly impaired replication in MDCK and Calu-3 cells, which was more pronounced with E23K. In ferret transmission experiments, all viruses transmitted to direct-contact and airborne-transmission animals, with only E23K+I38T viruses failing to infect 100% of animals by airborne transmission. E23G/K genotypes were predominantly stable during transmission events and through five passages in vitro. Thermostable PA–BXA interactions were weakened by E23G/K substitutions and further weakened when combined with I38T. In silico modeling indicated this was caused by E23G/K altering the placement of functionally important Tyr24 in the endonuclease domain, potentially decreasing BXA binding but at some cost to the virus. These data implicate E23G/K, alone or combined with I38T, as important markers of reduced BXM susceptibility, and such mutants could emerge and/or transmit among humans.

## Author summary

Baloxavir is a new and potent anti-influenza drug targeting essential functions of viral replication. Currently, the I38T polymerase acidic protein (PA) substitution is the major marker of reduced susceptibility and potential resistance to baloxavir, but the full baloxavir resistance profile remains unclear. Here, we demonstrated that PA E23G/K substitutions alone weaken baloxavir efficacy, but they also synergize with I38T to impair drug activity further. E23G/K substitutions are located close to the binding site of baloxavir and indirectly weaken key drug-binding interactions. This effect has some negative consequences for virus replication, but E23G/K viruses possess the capacity for airborne spread between naïve ferrets, the gold-standard model of human influenza transmission. Therefore, E23G/K viruses have the potential for community spread, which would adversely affect baloxavir clinical implementation. Our study supports ongoing surveillance for circulating human E23G/K viruses, and it may inform design of enhanced baloxavir-like drugs less susceptible to emergence of viral resistance.

## Introduction

Influenza A and B viruses are highly contagious respiratory pathogens that cause annual epidemics and occasional pandemics. Vaccines are effective preventative measures, but antiviral agents play an important adjunct role in influenza treatment and control. The two clinically approved classes of influenza antivirals are the neuraminidase (NA) inhibitors (NAIs), which target the viral NA protein and inhibit virus budding, and a polymerase acidic protein (PA) endonuclease inhibitor that targets the PA protein and viral transcription. Four NAIs have been approved in various countries [1], whereas only one endonuclease inhibitor (baloxavir marboxil, BXM) is currently clinically available [2]. Viral resistance can arise during treatment with either type of agent. The resistance landscape of the NAIs is well defined because these agents have been used for more than 20 years [2,3]. In contrast, BXM entered the market only recently (2018), and identification of molecular markers associated with reduced susceptibility to its active metabolite, baloxavir acid (BXA), is ongoing [2,4].

Currently, phenotypic tests defining BXA resistance profiles differ between laboratories, and fold-changes that signify BXA “resistance” are not fully established. Therefore, ongoing identification, surveillance, and determination of the fitness characteristics of viruses containing BXA-associated PA changes is critically important. Substitutions conferring reduced BXA susceptibility have been identified in the drug’s target binding site, the 200–amino acid PA endonuclease domain (PA_N_). PA residue 38 is a hotspot for substitutions (I38T/M/F/L/N/S), and I38T or I38X mixtures were identified early in laboratory studies [5,6], clinical trials [6–8], and household/close-contact transmission observations [9–11]. Non-I38X PA_N_ substitutions, including PA E23X, have resulted from BXM treatment of patients [6,12]. We also identified E23K, alone and combined with I38T, after serial passage with the BXA analogue RO-7 [5]. E23K was subsequently identified in baloxavir phase II/III trials and found to cause virus rebound [6,7,12], and also in multiple post-approval reports [9,11,13]. E23G was identified in a pediatric clinical cohort [6,7], a surveillance study of North American human viruses (2016–2017) [14], but seldom in post-BXA approval surveillance [7,15]. Both substitutions are noted as potential molecular determinants of reduced BXM susceptibility in product packaging [16]. However, polymorphisms at PA residue 23 are thus far rarely observed among human influenza viruses of various subtypes (S1 Table).

Transmission potential of viruses with reduced BXM susceptibility has been demonstrated in animal models [4], but largely limited to I38X substitutions. Clinical evidence suggests that E23X viruses possess the same transmission potential. An A(H1N1)pdm09 infection of a non–BXM-treated child yielded an E23K isolate with a 7-9-fold increase in the BXA EC_50_ [13]. A trial examining BXM prophylaxis after household influenza exposure identified five patients shedding E23K viruses; three of these viruses were E23K substituted before antiviral intervention [9]. The potential for E23K transmission was suggested by both studies, and although this raises the prospect of E23G/K viruses spreading, studies of E23G/K virus polymerase activity, growth kinetics in multiple virus backgrounds, transmission in animals, and structural mechanism(s) of resistance have not been performed. E23 is located near the BXA binding site within PA_N_ and, therefore, could influence both drug inhibition and critical viral functions. We hypothesized that E23G/K substitutions reduce the efficacy of BXA, but potentially at the cost of some replicative and/or transmission fitness. Furthermore, we sought to examine the potential for synergism of these substitutions with I38T and to understand the mechanism(s) underlying E23G/K-associated impairment of BXA activity.

## Results

### Effects of PA E23G/K and E23G/K+I38T substitutions on polymerase complex activity

**Fig 1:**
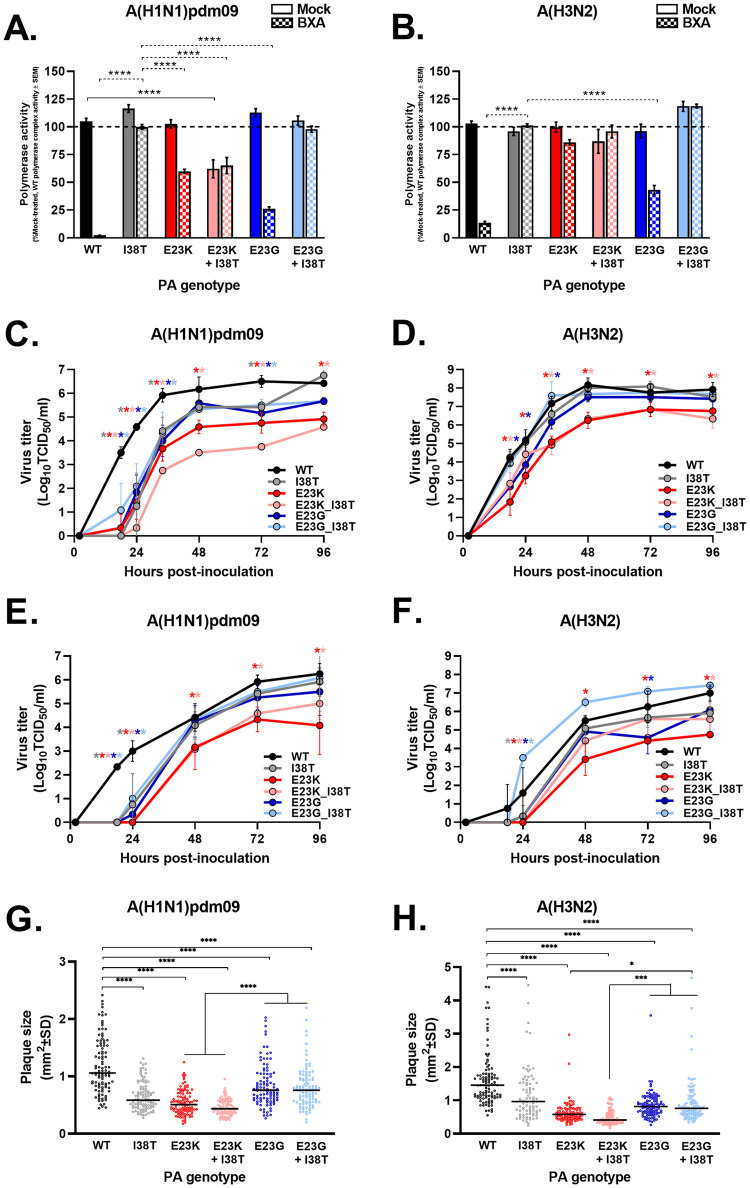
In vitro fitness of PA E23G/K influenza A viruses. Fitness profiles were defined in three assays. **A, B)** In the minireplicon assay, HEK293T cells were transfected with plasmids expressing virus polymerase complex proteins, the indicated PA mutant, a luciferase reporter of viral polymerase activity, and a β-galactosidase transfection control reporter plasmid. Cells were mock treated (with DMSO, solid bars) or treated with BXA (5 nM, checkered bars) 1 h before and 24 h after transfection. The polymerase activity luciferase output was normalized to the β-galactosidase signal for each data point then compared to the activity of mock-treated WT polymerase (100% activity) or I38T polymerase (100% BXA “resistance”). The data are representative of combined replicates (2–3 per experimental group) from five experiments ± SEM, **** *P* ≤ 0.001 vs. 0 nM treated (solid comparison lines) or BXA treated (dotted comparison lines), absence of a marker indicates no significant differences; Replication kinetics were determined in **C,D)** virus-inoculated MDCK cells (MOI: 0.001) and **E, F)** Calu-3 cells (MOI: 0.01). Supernatants were collected as indicated, and titers were determined by TCID_50_ assay in MDCK cells. The data are representative experiments of at least triplicate measures at each time point ± SD, * *P* ≤ 0.05 vs. WT, with the asterisks color coded to each virus; **G, H)** Plaque assays were used to measure the mean plaque diameter at 72–96 hpi in MDCK cells. The data are representative of two independent assays of six replicate wells of >87 plaques ± SD, * *P* ≤ 0.05, ** *P* ≤ 0.01, *** *P* ≤ 0.001, **** *P* ≤ 0.001.

The effects of E23G/K and E23G/K+I38T on polymerase complex activity were examined in the minireplicon reporter assay [17]. Polymerase activity in the absence of BXA inhibitor was normalized to wild-type (WT) complexes (100% activity) in the A(H1N1)pdm09 and A(H3N2) subtypes. E23G, E23K, and E23G+I38T did not significantly decrease polymerase activity compared to WT complexes, nor did I38T alone [18–20]. E23K+I38T complexes in the A(H1N1)pdm09 subtype significantly reduced polymerase activity [*P* ≤ 0.0001], reaching only 62% activity of the WT. While the same substitutions conferred only 87% the activity of WT in the A(H3N2) subtype, this was not statistically significant (Fig 1A and 1B).

Polymerase activity in the presence of 5 nM BXA, a concentration within the range of early reports of E23X-asssociated EC_50_ values [6,12,14,21], was normalized to the I38T complexes (100% BXA “resistant”). BXA significantly inhibited 87%–98% of WT complex activity [*P* ≤ 0.0001], demonstrating the strong susceptibility of those polymerases. E23G/K complexes were significantly inhibited [*P* ≤ 0.0001] in A(H1N1)pdm09 (40–74% activity inhibited) (Fig 1A). E23G was significantly inhibited [*P* ≤ 0.0001] in A(H3N2) (57% activity inhibited), and E23K activity was 14% inhibited, though the latter was not statistically significant (Fig 1B). E23G+I38T and E23K+I38T complexes of A(H3N2) origin were not BXA susceptible (Fig 1A and 1B). In A(H1N1)pdm09, E23K+I38T complexes showed approximately 35% inhibition by BXA, but this reduced activity was equivalent to the maximum polymerase output in the absence of drug (Fig 1A), reflecting the impairment caused by this dual substitution. Therefore, E23G/K substitutions have modest, if any, adverse effects on influenza A virus polymerase complex activity. E23G complexes are more susceptible to BXA than are E23K complexes, but BXA susceptibility is most reduced when combined with I38T.

### Rescue of PA E23G/K and E23G/K+I38T viruses and BXA susceptibility phenotypes

To define BXA susceptibility phenotypes, we rescued A/California/04/2009 [A(H1N1)pdm09] and A/Texas/71/2017 [(A(H3N2)] viruses with E23G/K or dual E23G/K+I38T substitutions and determined 50% effective concentrations (EC_50_s) by plaque reduction assays (Table 1). A >3-fold increase in EC_50_ relative to WT has been conditionally proposed as a threshold for baloxavir ‘resistance’ [2,12,14].

**Table 1:** Influenza A(H1N1)pdm09 and A(H3N2) viruses used in this study and their susceptibility to BXA.

Virus	Abbreviation	PA genotype^a^	BXA susceptibility in the plaque reduction assay		BXA susceptibility in the minireplicon assay	
			(EC_50_ ± SEM, nM)^b^	Fold increase over WT EC_50_	(EC_50_ ± SEM, nM)^c^	Fold increase over WT EC_50_
rg-A/California/04/2009 (H1N1)pdm09	A(H1N1)pdm09	WT	0.22 ± 0.06	−	0.90 ± 0.07	−
		I38T	18.30 ± 2.80	83	160.40 ± 17.00	178
		E23K	2.80 ± 0.71	13	7.23 ± 0.18	8
		E23K+I38T	48.30 ± 16.50	220	1092.32 ± 338.41	1214
		E23G	1.63 ± 0.40	7	2.60 ± 0.18	3
		E23G+I38T	30.42 ± 5.82	138	678.32 ± 116.53	754
rg-A/Texas/71/2017 (H3N2)	A(H3N2)	WT	0.90 ± 0.60	−	1.92 ± 0.17	−
		I38T	69.83 ± 21.62	78	110.00 ± 7.05	57
		E23K	5.04 ± 1.20	6	20.23 ± 1.42	11
		E23K+I38T	401.73 ± 117.70	446	897.43 ± 116.82	467
		E23G	2.12 ± 0.68	2	5.00 ± 0.19	3
		E23G+I38T	131.10 ± 48.71	146	553.00 ± 113.79	288

^a^ Amino acid identity at PA residue 23 and/or 38, introduced by reverse genetics (rg).

^b^ Reduction in plaque formation (50–100 PFU/well) in inoculated MDCK cells at 72 or 96 hpi. Average values from four independent dose–response curves are presented ± the standard error of the mean (SEM).

^c^ Reduction in the ratio of luciferase reporter activity (RFU) to beta-galactosidase activity (*A*_415_) in HEK293T cells transfected with PB1, PB2, PA, NP influenza virus genes, and assayed at 24 hr post-transfection. Cells were pre-treated (1 hr) and post-treated (24 hr) with BXA. Average values from 3 to 6 independent dose–response curves are presented ± the standard error of the mean (SEM).

The EC_50_ value of WT A(H1N1)pdm09 virus 0.22 nM, while the EC_50_ value of the A(H3N2) virus was 0.90 nM. The dominant BXA resistance marker I38T yielded EC_50_ values of 18.30 nM and 69.83 nM for A(H1N1)pdm09 and A(H3N2) (a >78-fold increase relative to WT) respectively, and consistent with previous reports [22]. E23G/K EC_50_ values ranged from 1.63 to 5.04 nM, with the E23K substitution (a 6- to 13-fold increase relative to WT EC_50_) consistently conferring greater BXA resistance than E23G (a 2- to 7-fold increase relative to WT). Combining I38T with E23G or E23K dramatically increased BXA EC_50_ values to 30.42–401.73 nM, which were greater than the sum of the EC_50_ values from the individual substitutions. Viruses with E23K+I38T were more resistant to BXA (a 220- to 446-fold increase relative to WT) than were those with E23G+I38T (138- to 146-fold increases relative to WT), and a trend towards higher EC_50_ values was observed with the A(H3N2) viruses (Table 1). We also determined EC_50_ values in the minireplicon assay, where values were generally higher compared to the plaque reduction assay. WT EC_50_ values were 0.90–1.92 nM. I38T EC_50_ values were 110.00–160.40 nM (a 57- to 178-fold increase relative to WT), E23K EC_50_ values were 7.23–20.23 nM (an 8- to 11-fold increase relative to WT), and E23G EC_50_ values were 2.60–5.00 nM (a 3-fold increase relative to WT). The combination of E23G/K with I38T once again dramatically increased BXA EC_50_ values to 553.00–1092.32 nM (a 288- to 1214- fold increase relative to WT), and these values remained greater than the sum of the EC_50_ values from the individual substitutions. Amongst both assays, E23K-containing viruses were more resistant to BXA than E23G-containing viruses, consistent with the trend observed in the minireplicon assays (Fig 1A and 1B).

### Replication kinetics of PA E23G/K and E23G/K+I38T viruses in vitro

We next examined the replication capacity of E23G/K and E23G/K+I38T viruses in MDCK cells. All E23G/K single- or dual-substituted viruses replicated productively, but the kinetics differed between substitutions and subtypes. In A(H1N1)pdm09 viruses, all substitutions resulted in replication being slower than that of WT virus until 48–72 hours post infection (hpi) [*P* ≤ 0.05]. By 96 hpi, the titers of E23G-containing viruses did not differ significantly from those of WT virus, whereas the titers of E23K-containing viruses continued to lag behind those of WT (Fig 1C) [*P* ≤ 0.05]. Replication of E23G/K and E23K+I38T A(H3N2) viruses was slower than that of WT virus until 48 hpi [*P* ≤ 0.05]. From 48 hpi onward, there was no significant difference between the titers of E23G-containing viruses and those of WT, whereas the titers of E23K viruses again lagged (Fig 1D) [*P* ≤ 0.05]. Plaque diameters for E23K viruses were 51%–62% smaller than those for WT virus, and plaque diameters for E23G viruses were 29%–42% smaller than those for WT. Adding a I38T decreased the E23K plaque diameters by an additional 7%–10% and the E23G plaque diameters by an additional 1%–7%. Continuing the trend, A(H1N1)pdm09 viruses with E23K substitution alone or in combination with I38T produced plaques 31%–46% smaller than those produced by viruses with E23G (Fig 1G), whereas A(H3N2) viruses with E23K+I38T produced plaques 34%–46% smaller than those produced by viruses with E23G (Fig 1H).

Replication capacity of E23G/K and E23G/K+I38T viruses were also determined human bronchial epithelia-derived Calu-3 cells that support influenza virus replication and share characteristics of the human proximal lower airway. Similar to MDCKs, E23G-containing viruses seldom exhibited titers lower that WT past 24 hpi. In contrast, E23K-containing viruses often produced statistically lower titers than WT, as late as 96 hpi (Fig 1E and 1F) [*P* ≤ 0.05].

Overall, and with respect to replication kinetics and plaque size, E23K had a greater negative impact on in vitro fitness than did E23G, and this was consistent for the single and dual substitutions in both subtypes.

### Genetic stability of PA E23G/K and E23G/K+I38T viruses

E23X-substituted genotypes are rarely observed in circulating influenza viruses (S1 Table) but have been detected in BXM-treated patients and have been implicated in household transmission. To understand the stability of E23G/K substitutions, viruses were passaged five times in MDCK cells without BXA. Passage 0 (P0) and P5 viruses were deep sequenced and variant frequencies were determined in the PA gene. In the P0 stocks, 100% of the viruses possessed the expected parental, reverse genetics derived PA genotype. The PA of P5 A(H3N2) viruses remained unchanged from the inoculated genotype. Reversion in A(H1N1)pdm09 viruses to the parental PA genotype was uncommon, but one virus replicate each had ~12% T→I reversion in the I38T virus group, ~21% G→E reversion in the E23G virus group, and ~12% G→E reversion in the E23G+I38T virus group (Table 2). Therefore, the E23K substitution remains genetically stable through cell passage in the absence of BXA pressure, while E23G reverted to parent PA genotype in minor proportions of the viral quasi-species and occurred in only one of two replicate passaged viruses.

**Table 2:** Genotype stability of influenza A(H1N1)pdm09 and A(H3N2) viruses passaged in the absence of baloxavir.

Virus	Input PA genotype^a^	Passage no.^b^	% PA 23 AA identity^c^			% PA 38 AA identity^c^	
			E23	K23	G23	I38	T38
rg-A/California/04/2009 (H1N1)pdm09	E23-WT	P0	100.0	-	-	100.0	-
		P5^1^	100.0	-	-	100.0	-
		P5^2^	100.0	-	-	100.0	-
	T38	P0	100.0	-	-	-	100.0
		P5^1^	100.0	-	-	11.6	88.4
		P5^2^	100.0	-	-	-	100.0
	K23	P0	-	100.0	-	100.0	-
		P5^1^	-	100.0	-	100.0	-
		P5^2^	-	100.0	-	100.0	-
	K23+T38	P0	-	100.0	-	-	100.0
		P5^1^	-	100.0	-	-	100.0
		P5^2^	-	100.0	-	-	100.0
	G23	P0	-	-	100.0	100.0	-
		P5^1^	-	-	100.0	100.0	-
		P5^2^	20.8	-	79.2	100.0	-
	G23+T38	P0	-	-	100.0	-	100.0
		P5^1^	-	-	100.0	-	100.0
		P5^2^	12.0	-	88.0	-	100.0
rg-A/Texas/71/2017 (H3N2)	E23-WT	P0	100.0	-	-	100.0	-
		P5^1^	100.0	-	-	100.0	-
		P5^2^	100.0	-	-	100.0	-
	T38	P0	100.0	-	-	-	100.0
		P5^1^	100.0	-	-	-	100.0
		P5^2^	100.0	-	-	-	100.0
	K23	P0	-	100.0	-	100.0	-
		P5^1^	-	100.0	-	100.0	-
		P5^2^	-	100.0	-	100.0	-
	K23+T38	P0	-	100.0	-	-	100.0
		P5^1^	-	100.0	-	-	100.0
		P5^2^	-	100.0	-	-	100.0
	G23	P0	-	-	100.0	100.0	-
		P5^1^	-	-	100.0	100.0	-
		P5^2^	-	-	100.0	100.0	-
	G23+T38	P0	-	-	100.0	-	100.0
		P5^1^	-	-	100.0	-	100.0
		P5^2^	-	-	100.0	-	100.0

^a^ Amino acid identity at PA residue 23 and/or 38, introduced by reverse genetics (rg).

^b^ Passage (P) 0 and P5 were sequenced. Superscript numbers indicate one of two separate passaged virus samples.

^c^ Amino acid variant calling was performed in CLC Workbench with variant frequency cut-off of 5% with ≥ 1000 reads/sample or 10% with <1000 reads/sample.

### Transmissibility of PA E23G/K and E23G/K+I38T viruses in ferrets

BXM-related fitness deficiencies in vitro do not necessarily predict a lack of fitness in vivo [19,23–26]. Therefore, we tested direct- (DC) and airborne-contact (AC) routes of A(H1N1)pdm09 virus transmission in ferrets, the gold-standard model of human influenza virus spread [27].

**Fig 2:**
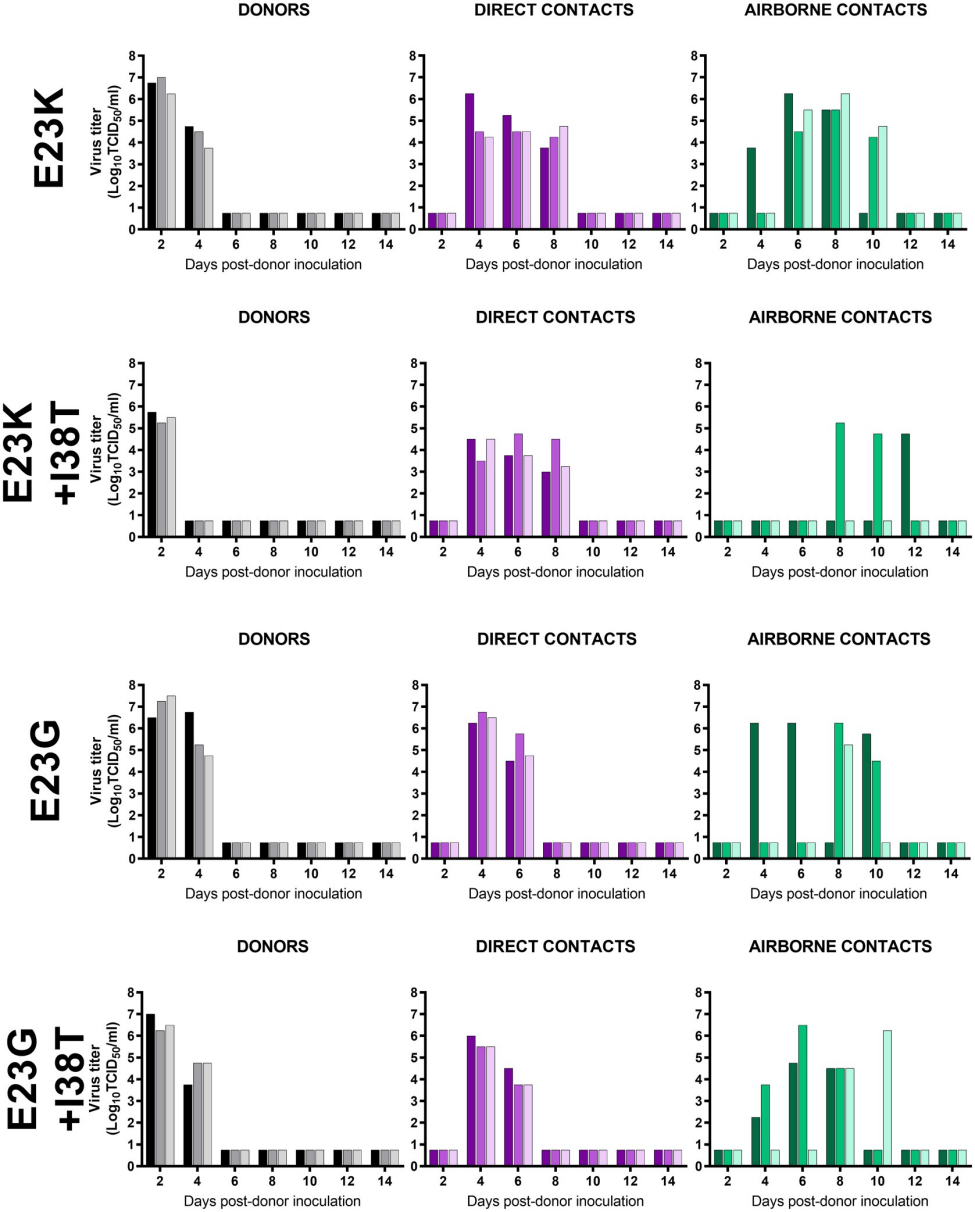
Transmissibility of PA E23G/K influenza A viruses in ferrets. Donor ferrets (n = 3, black/gray) were inoculated intranasally with the indicated PA-substituted virus. Twenty-four hours later, direct contact animals (n = 3, shades of purple, each in the same cage as the respective donor) and airborne contact animals (n = 3, shades of green, in an adjacent cage with an air-permeable barrier that excluded physical contact) were introduced. Virus titers (in Log_10_TCID_50_/mL; limit of detection: 0.75 Log_10_TCID_50_/mL) were determined in nasal washes collected at the indicated timepoints and titrated in MDCK cells. Color-matched bars represent the virus shed by an individual animal over time.

**Fig 3:**
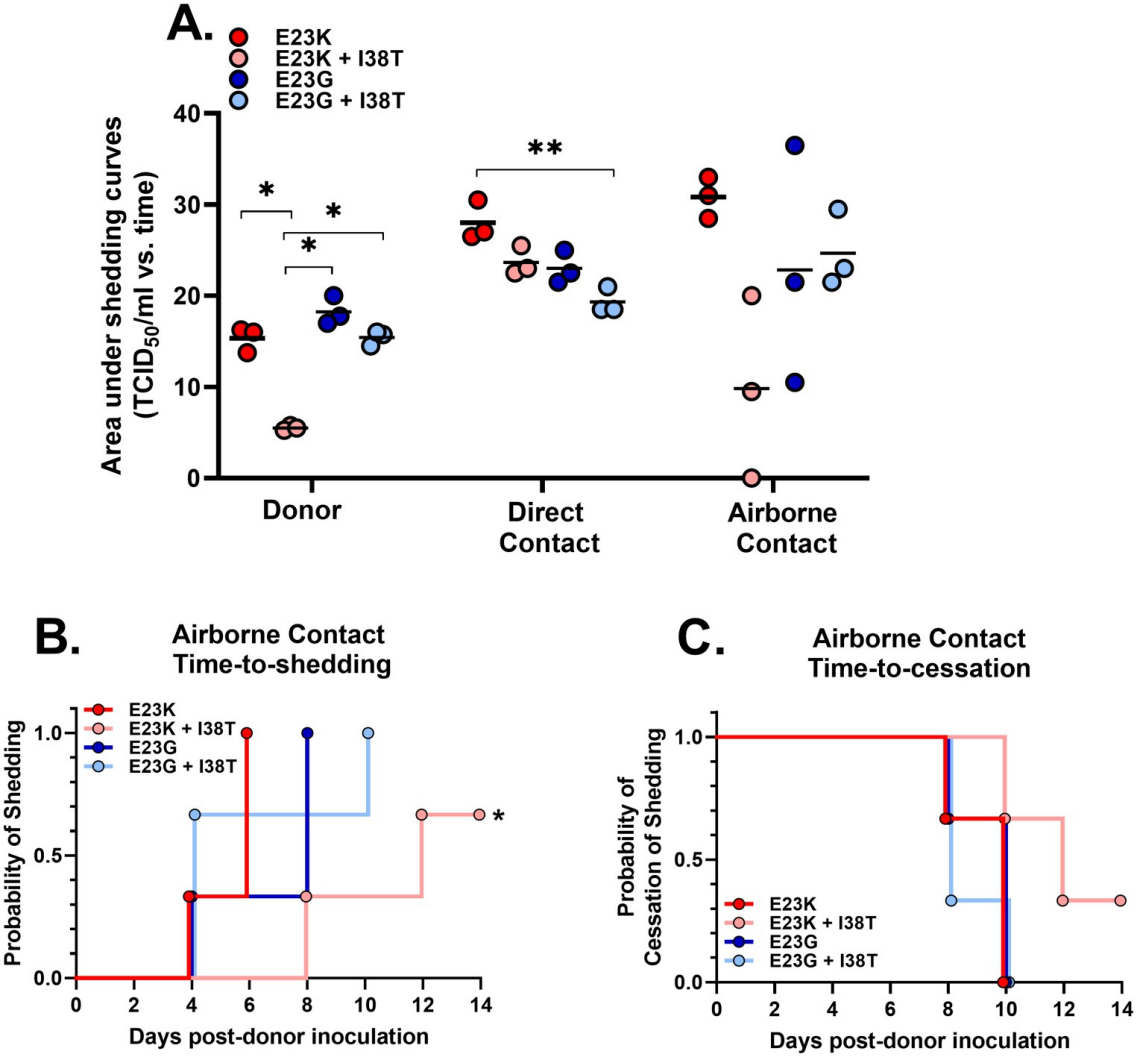
Shedding parameters of PA E23G/K influenza A viruses in ferrets. A) The nasal washes described in Fig 2 were subjected to area-under-the-curve (AUC) analysis by contact type. * *P* ≤ 0.03, ** *P* ≤ 0.01 **B, C)** Airborne contact nasal sampling was analyzed by Kaplan–Meier curves and the log-rank test to estimate the time-to-events, including the onset of shedding and the cessation of shedding. * *P* < 0.03 vs. E23K.

All viruses, regardless of substitution, were shed by three of three donors until 2–4 days post infection (dpi). The area under the curve (AUC_0–14_) was used to quantify virus shedding. For donor ferrets inoculated with E23K+I38T virus, the shedding AUC was approximately one-third of that for animals inoculated with E23K virus (the AUC_0-14_s were 5.8 and 16.0, respectively) and was significantly smaller than that for animals infected with E23G or E23G+I38T virus. All viruses transmitted to three of three DC animals. One less shedding timepoint was noted for E23G-containing viruses, and E23G+I38T DC ferrets had a smaller shedding AUC when compared to ferrets infected with E23K alone. Finally, all viruses exhibited airborne transmission, although for the E23K+I38T virus, only two of three AC ferrets shed virus and the AUC values were not significantly different from those for other groups (Figs 2 and 3A).

To investigate further the impaired airborne transmission of E23K+I38T viruses, the time-to-shedding (TTS_hed_) and/or time-to-cessation of shedding (TTC_ess_) of AC animals were plotted with univariate comparisons. The TTS_hed_ curves for E23K+I38T viruses were significantly different (*P* < 0.03) from those for E23K viruses, and the median TTS_hed_s were 12 days and 6 days, respectively (Fig 3B). No significant differences in TTC_ess_ were observed among AC animals inoculated with E23K or E23K+I38T viruses (Fig 3C). The TTS_hed_ for E23G+I38T viruses was qualitatively longer than that for E23G-only viruses, but not significantly so, nor was there any difference in the TTC_ess_ curves (Fig 3B and 3C).

Most animals displayed one or more respiratory symptoms (sneezing, coughing, congested breathing) during infection, and these symptoms were similar regardless of the virus genotype. This trend generally continued for clinical events (lethargy, fever, anorexia), with the exception that E23K+I38T DC and AC ferrets and two of three E23K+I38T donors displayed no clinical symptoms. All animals, regardless of contact type or the virus inoculum, seroconverted to homologous WT virus (hemagglutination inhibition titers of 1:80–1:2560), including the E23K+I38T AC animal that shed no virus and displayed no clinical disease (S2 Table).

Overall, E23G/K and E23G/K+I38T viruses retained transmissibility in ferrets by the DC and AC routes. Dual-substituted E23K+I38T virus exhibited some attenuation characterized by incomplete airborne transmission, decreased incidence of clinical disease, and increased time-to-shedding among AC animals.

### Genetic stability of PA E23G/K and E23G/K+I38T viruses during ferret transmission

To investigate whether E23G/K-containing viruses reverted to the generally fitter E23 or I38 genotypes during transmission, we amplified PA_N_ segment sequences from ferret nasal washes with a positive virus titer and subjected them to deep sequencing with analysis of variance at PA 23 and PA 38. Virus inocula were found to be 100% of the intended genotype. Reversion events were rare, and E23G/K substitutions with or without I38T were remarkably stable upon DC or airborne transmission. No reversions to WT were observed in donors or in DC transmission events for any virus. Among the AC ferrets, one of three E23K-inoculated animals displayed a 13.3% K→E reversion beginning 8 dpi, which increased to 18.3% K→E at 10 dpi. No other reversions to the E23 genotype were observed in AC animals, regardless of the inoculated virus. However, one E23G-inoculated AC ferret displayed a 26.6% I38→T substitution at 8 dpi, although the sample quality was poor (<1000 reads of coverage) and this genotype was not detected in the subsequent 10 dpi nasal wash (Table 3). Therefore, successful transmission of E23G/K viruses is not the result of reversion to WT.

**Table 3:** Genotype of A(H1N1)pdm09 viruses isolated from ferret nasal washes.

PA genotype of virus inoculum^a^	Route of transmission^b^	Days post-donor inoculation	% PA 23 AA identity^c^			% PA 38 AA identity^c^	
			E23	K23	G23	I38	T38
E23K	Donor #1	2	-	100.0	-	100.0	-
		4	-	100.0	-	100.0	-
	Donor #2	2	-	100.0	-	100.0	-
		4	-	100.0	-	100.0	-
	Donor #3	2	-	100.0	-	100.0	-
		4	-	100.0	-	100.0	-
	DC #1	4	-	100.0	-	100.0	-
		6	-	100.0	-	100.0	-
		8	-	100.0	-	100.0	-
	DC #2	4	-	100.0	-	100.0	-
		6	-	100.0	-	100.0	-
		8	-	100.0	-	100.0	-
	DC #3	4	-	100.0	-	100.0	-
		6	-	100.0	-	100.0	-
		8	-	100.0	-	100.0	-
	AC #1	4	-	100.0	-	100.0	-
		6	-	100.0	-	100.0	-
		8	-	100.0	-	100.0	-
	AC #2	6	-	100.0	-	100.0	-
		8	13.3	86.7	-	100.0	-
		10	18.3	81.7	-	100.0	-
	AC #3	6	-	100.0	-	100.0	-
		8	-	100.0	-	100.0	-
		10	-	100.0	-	100.0	-
	Donor inoculum^d^	N/A	-	100.0	-	100.0	-
E23K+I38T	Donor #1	2	-	100.0	-	-	100.0
	Donor #2	2	-	100.0	-	-	100.0
	Donor #3	2	-	100.0	-	-	100.0
	DC #1	4	-	100.0	-	-	100.0
		6	-	100.0	-	-	100.0
		8	-	100.0	-	-	100.0
	DC #2	4	-	100.0	-	-	100.0
		6	-	100.0	-	-	100.0
		8	-	100.0	-	-	100.0
	DC #3	4	-	100.0	-	-	100.0
		6	-	100.0	-	-	100.0
		8	-	100.0	-	-	100.0
	AC #1	12	-	100.0	-	-	100.0
	AC #2	8	-	100.0	-	-	100.0
		10	-	100.0	-	-	100.0
	Donor inoculum	N/A	-	100.0	-	-	100.0
E23G	Donor #1	2	-	-	100.0	100.0	-
		4	-	-	100.0	100.0	-
	Donor #2	2	-	-	100.0	100.0	-
		4	-	-	100.0	100.0	-
	Donor #3	2	-	-	100.0	100.0	-
		4	-	-	100.0	100.0	-
	DC #1	4	-	-	100.0	100.0	-
		6	-	-	100.0	100.0	-
	DC #2	4	-	-	100.0	100.0	-
		6	-	-	100.0	100.0	-
	DC #3	4	-	-	100.0	100.0	-
		6	-	-	100.0	100.0	-
	AC #1	4	-	-	100.0	100.0	-
		6	-	-	100.0	100.0	-
		8	-	-	100.0	73.4	26.6
		10	-	-	100.0	100.0	-
	AC #2	8	-	-	100.0	100.0	-
		10	-	-	100.0	100.0	-
	AC #3	8	-	-	100.0	100.0	-
		10	-	-	100.0	100.0	-
	Donor inoculum	N/A	-	-	100.0	100.0	-
E23G+I38T	Donor #1	2	-	-	100.0	-	100.0
		4	-	-	100.0	-	100.0
	Donor #2	2	-	-	100.0	-	100.0
		4	-	-	100.0	-	100.0
		6	-	-	100.0	-	100.0
	Donor #3	2	-	-	100.0	-	100.0
		4	-	-	100.0	-	100.0
	DC #1	4	-	-	100.0	-	100.0
		6	-	-	100.0	-	100.0
	DC #2	4	-	-	100.0	-	100.0
	DC #3	4	-	-	100.0	-	100.0
		6	-	-	100.0	-	100.0
	AC #1	4	-	-	100.0	-	100.0
		6	-	-	100.0	-	100.0
		8	-	-	100.0	-	100.0
	AC #2	4	-	-	100.0	-	100.0
		6	-	-	100.0	-	100.0
		8	-	-	100.0	-	100.0
	AC #3	8	-	-	100.0	-	100.0
		10	-	-	100.0	-	100.0
	Donor inoculum	N/A	-	-	100.0	-	100.0

^*a*^ Amino acid identity at PA residue 23 and/or 38, introduced by reverse genetics (rg).

^*b*^ Donors were directly inoculated and paired with direct (DC) or airborne contacts (AC) as described in the Materials and Methods section.

^*c*^ Amino acid variant calling was performed in CLC Workbench with variant frequency cut-off of 5% with ≥ 1000 reads/sample or 10% with <1000 reads/sample.

^*d*^ Virus inocula were resequenced and verified as being 100% of the intended amino acid identity.

### Effects of PA E23G/K substitutions on PA_N_–BXA interactions

**Fig 4:**
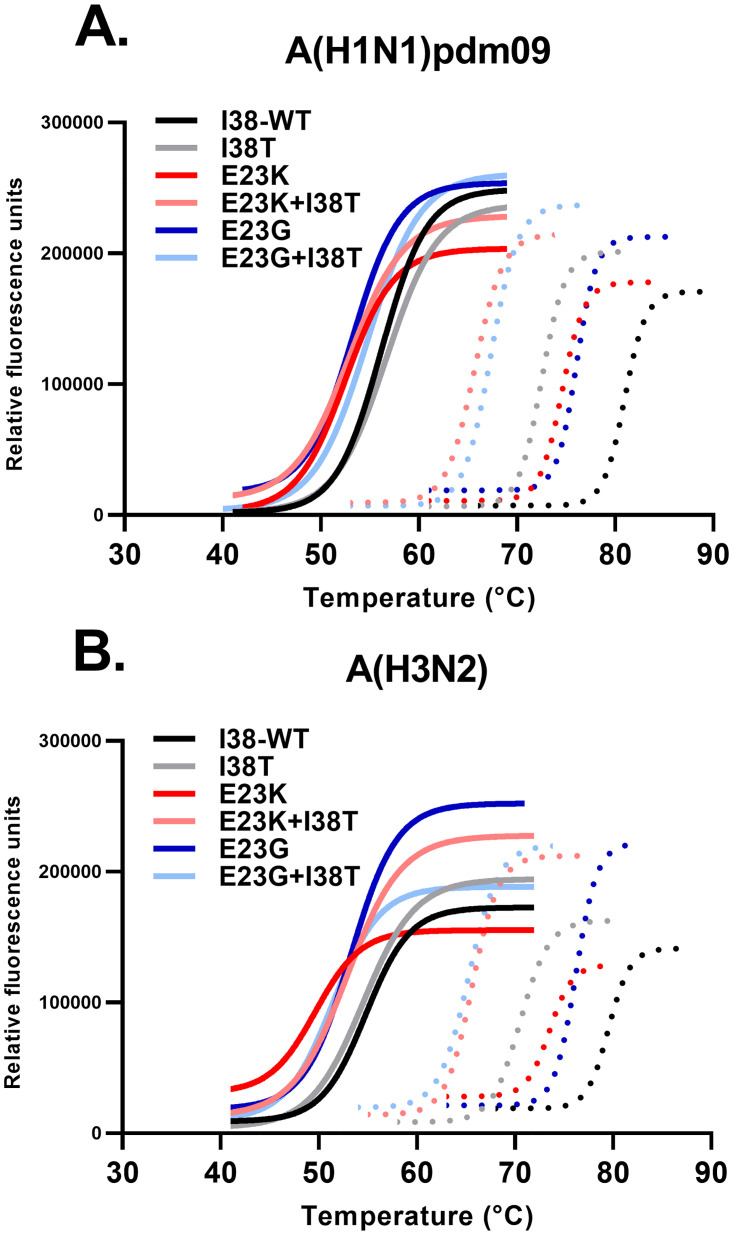
Thermal stability of BXA-rPA_N_ with E23G/K substitutions. The thermal stability of BXA-rPA_N_ complexes was assessed in a modified thermofluor assay. rPA_N_ (2 μM) from the **A)** A(H1N1)pdm09 or the **B)** A(H3N2) subtype was incubated with vehicle (DMSO, solid lines) or BXA (37 μM, dotted lines), along with SYPRO Orange and progressively heated at 1°C/min. The emitted SYPRO signal was read at each step. Thermal melt curves were fitted by the Boltzman sigmoidal equation and trimmed at the extreme of each fitted curve. The curves were used to estimate the 50% melting temperatures (T_m_) and the change between the curves for vehicle treatment and BXA treatment (ΔT_m_) (Table 4). The results are representative of five independent experiments with triplicate replicates for each sample.

**Fig 5:**
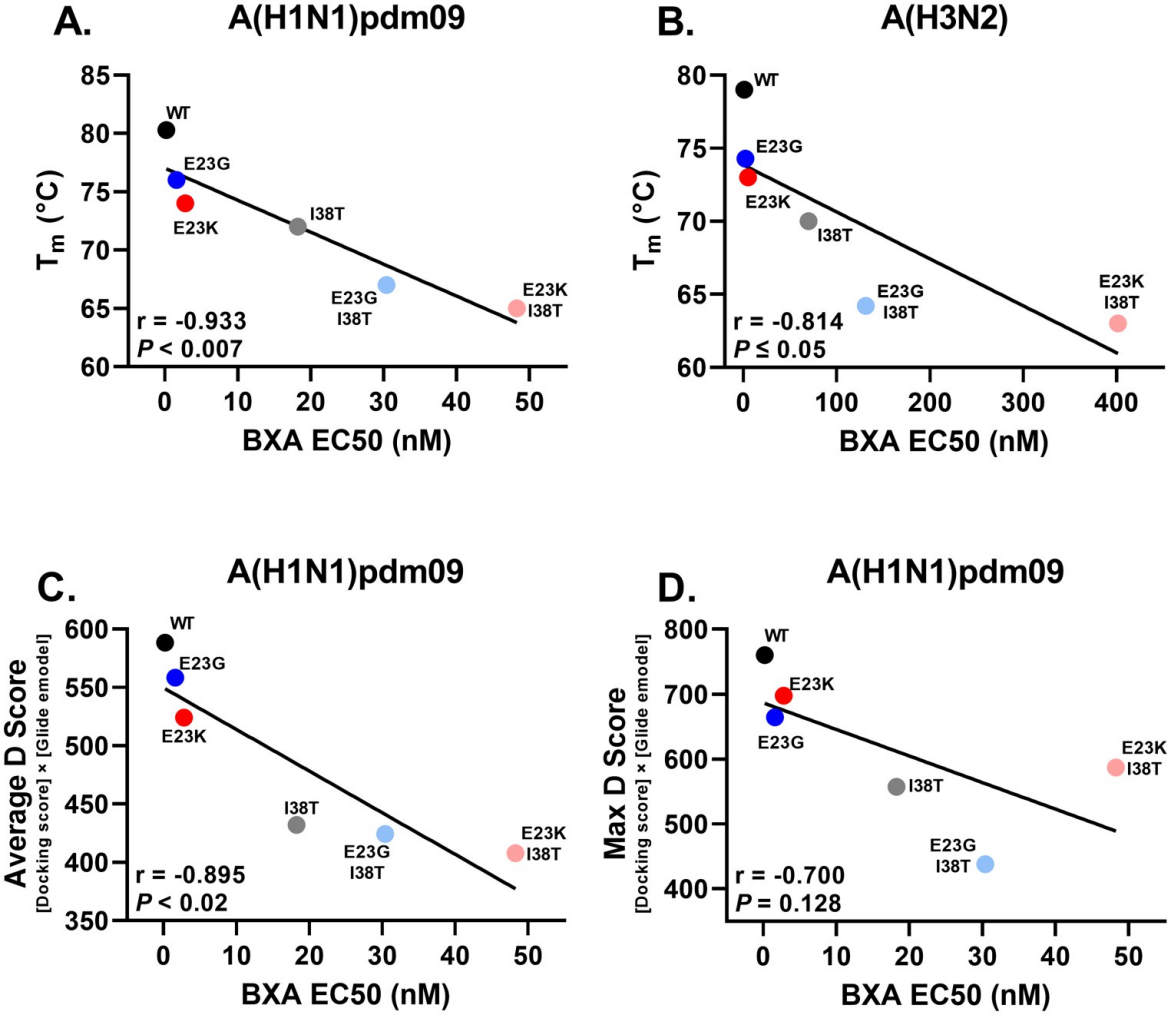
Correlation between the rPA_N_ thermal stability or the molecular docking score and the BXA EC_50_. Pearson correlation analysis of **A, B)** rPA_N_-BXA thermal stable T_m_ values vs. BXA EC_50_s, or **C, D)** in silico derived docking scores vs. BXA EC_50_s. The virus, docking mode, Pearson correlation coefficient, and statistical significance is listed in each panel.

The ability of BXA (37 μM) to bind and thermostabilize recombinant E23G/K or E23G/K+I38T PA_N_ was examined by a modified thermofluor assay [6]. The 50% protein melting temperatures (T_m_) and the changes in T_m_ (ΔT_m_) between samples treated with vehicle (DMSO) and those treated with BXA were derived from melt curves (30–99°C). Control reactions with A(H1N1)pdm09 or A(H3N2) WT or I38T vehicle-treated PA_N_ resulted in T_m_s of 54–55°C, which shifted markedly when WT PA_N_ was BXA-treated (to 79–80°C; ΔT_m_: 25°C). The temperature shift was smaller when I38T PA_N_ was BXA-treated (70–72°C; ΔT_m_: 16–17°C), implying impaired formation of protein–drug complexes. The thermal stabilities of vehicle-treated E23G/K-containing proteins were similar, ranging from 48°C-55°C, but they shifted variably upon BXA treatment. E23G/K alone produced the highest T_m_s (with more stable protein–drug complexes) at 73–76°C, followed by I38T alone at 70–72°C and then E23G/K+I38T at 63–67°C, resulting in a T_m_ pattern of WT > E23G > E23K > I38T > E23G+I38T > E23K+I38T (Fig 4A and 4B and Table 4 and S3 Table). These patterns were consistent among A(H1N1)pdm09 and A(H3N2) proteins. Therefore, proteins with single E23G/K substitutions form stronger BXA interactions than those with I38T alone or E23G/K+I38T, and this is functionally represented by a robust negative correlation to the hierarchies of BXA EC_50_ values (Pearson R values = −0.814 for A(H3N2) and −0.933 for A(H1N1)pdm09, *P* < 0.05) (Fig 5A and 5B).

**Table 4:** Thermal stabilization of E23X E23G/K and/or I38T-substituted PA_N_ by BXA.

PA_N_ origin^a^	PA_N_ genotype	Thermal stabilization parameters		
		T_m_ (°C)^b^ without BXA	T_m_ (°C)^b^ with BXA	ΔT_m_ (°C)^c^
A(H1N1)pdm09	WT	55.1 ± 0.2	80.3 ± 0.1	25.3 ± 0.2
	I38T	55.3 ± 0.2	72.0 ± 0.1	16.5 ± 0.3
	E23K	51.2 ± 0.3	74.0 ± 0.1	23.0 ± 0.3
	E23K+I38T	52.0 ± 0.4	65.0 ± 0.1	13.0 ± 0.4
	E23G	52.4 ± 0.3	76.0 ± 0.1	23.1 ± 0.3
	E23G+I38T	54.0 ± 0.3	67.0 ± 0.1	13.1 ± .3
A(H3N2)	WT	54.0 ± 0.3	79.0 ± 0.1	25.0 ± 0.3
	I38T	54.0 ± 0.1	70.0 ± 0.1	16.1 ± 0.2
	E23K	49.0 ± 0.4	73.0 ± 0.1	24.0 ± 0.4
	E23K+I38T	48.2 ± 1.0	63.0 ± 0.6	14.3 ± 0.4
	E23G	51.4 ± 0.2	74.3 ± 0.3	23.0 ± 0.2
	E23G+I38T	50.4 ± 0.2	64.2 ± 0.1	14.0 ± 0.3

^*a*^ Recombinant influenza A, N-terminal endonuclease domain (1.3 μg, 2 μM)

^b^ 50% protein melting temperatures derived from five independent melt curves (30–99°C) by modified thermofluor assay ± SEM.

^c^ Change in Tm between samples treated without BXA (DMSO) and those treated with BXA (37 μM).

* A higher T_m_ value indicates more successful/robust protein-drug complex formation.

### In silico modeling of PA E23G/K–BXA interactions

We employed an induced-fit docking molecular dynamics (IFD-MD) workflow [28] incorporating multiple computer modeling techniques to predict BXA–PA_N_ binding modes and understand the mechanisms by which E23G/K reduced BXA susceptibility. E23G/K point mutations and BXA placement were introduced into existing A(H1N1)pdm09 PA_N_ structures. The BXA–PA_N_ average and best-pose docking scores were always higher (indicating greater potential for interaction) for WT and E23G/K than for I38T and E23G/K+I38T. The average Glide eModel scores precisely recapitulated the patterns of decreasing affinity (S4 Table) predicted by thermostability assays (Table 4) and increasing plaque EC_50_s (Table 1). Additionally, there was a negative correlation (Pearson R value = −0.895, *P* < 0.02) between the average D-score and EC_50_ values (Fig 5C and 5D). Overall, the scores derived from docking poses generally predict decreased affinity of PA_N_ with E23G/K, which further decreases with I38T addition.

**Fig 6:**
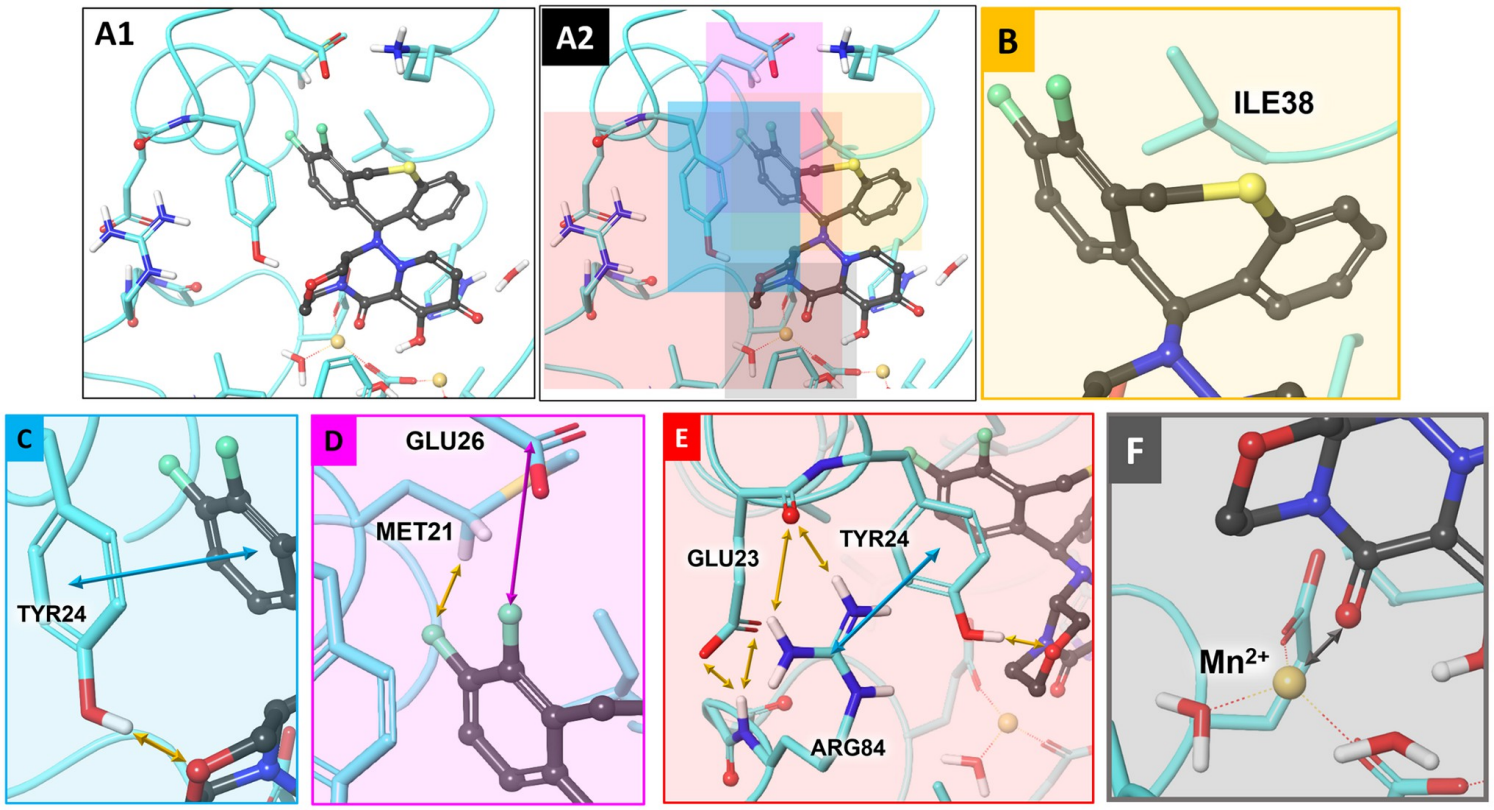
Predicted poses describing key BXA-rPA_N_ interactions of WT A(H1N1)pdm09. **A1, A2)** Overall view of the BXA binding interface. **B)** Multiple hydrophobic contacts between the S-containing tricyclic system and the Ile38 residue. **C)** A hydrogen bond (HB) between the phenol H-atom of the Tyr24 residue and the morpholine O atom of BXA, and π-stacking between the Tyr24 phenyl ring and the fluorinated phenyl ring of BXA. **D)** A T-shaped orthogonal multipolar interaction between the second fluorine at the o-position and the carboxyl C atom of Glu26 (COO). **E)** The HB-stabilized cluster of Glu23 and Arg84 that co-stabilizes π-stacking and/or π-cation interaction between Arg84 and Tyr24. **F)** BXA coordination by Mn^2+^ within the lower BXA tricyclic ring. Yellow arrows: hydrogen bonds; blue arrows: π-stacking interaction; pink arrows: halogen bonding; gray arrows: metal–ligand coordinating bond; dark-gray balls and sticks: BXA pose (pdb: 6FS6).

The following supramolecular interface (Fig 6A and 6A2) supports the different BXA binding affinities for PA_N_ with E23G/K or I38T substitutions: For WT PA_N_, there are 1) crucial hydrophobic contacts between the -CH2CH3 group of Ile38 and the two aromatic rings (6,11-dihydrodibenzo[b,e]thiepine) of BXA (Fig 6B); 2) hydrogen bonding (HB) between the -OH group of Tyr24 and the BXA morpholine oxygen, as well as inclined parallel-displaced stacking between the fluorinated BXA ring and Tyr24 (Fig 6C); 3) a weak HB between the fluorine atom at the p-position and the Cα proton of Met21 (Fig 6D); 4) a T-shaped orthogonal multipolar interaction between the second fluorine at the o-position and the carboxyl C atom of Glu26 (Fig 6D); and 5) two HBs between the Glu23 carbonyl O atom and Arg84 (guanidine group) and two HBs between the -NH- moiety of the Arg84 peptide stalk and the (COO-) of the Glu23 residue (Fig 6E). Importantly, the HB-stabilized Glu23 and Arg84 cluster maintains the conformation of the–(CH2)2- fragment of the Arg84 side chain (Cβ and Cγ). This conformation favors, first, the good π-stacking interaction between the Tyr24 phenyl ring and Arg84 (guanidine group) and, subsequently, good positioning of the Tyr24 -OH group for HB contact with the BXA morpholine oxygen and for π-stacking between BXA (the tricyclic moiety) and Tyr24. Additionally, BXA is coordinated by an Mn^2+^ ion within the amide carbonyl O atom of the lower tricyclic ring (Fig 6F).

**Fig 7:**
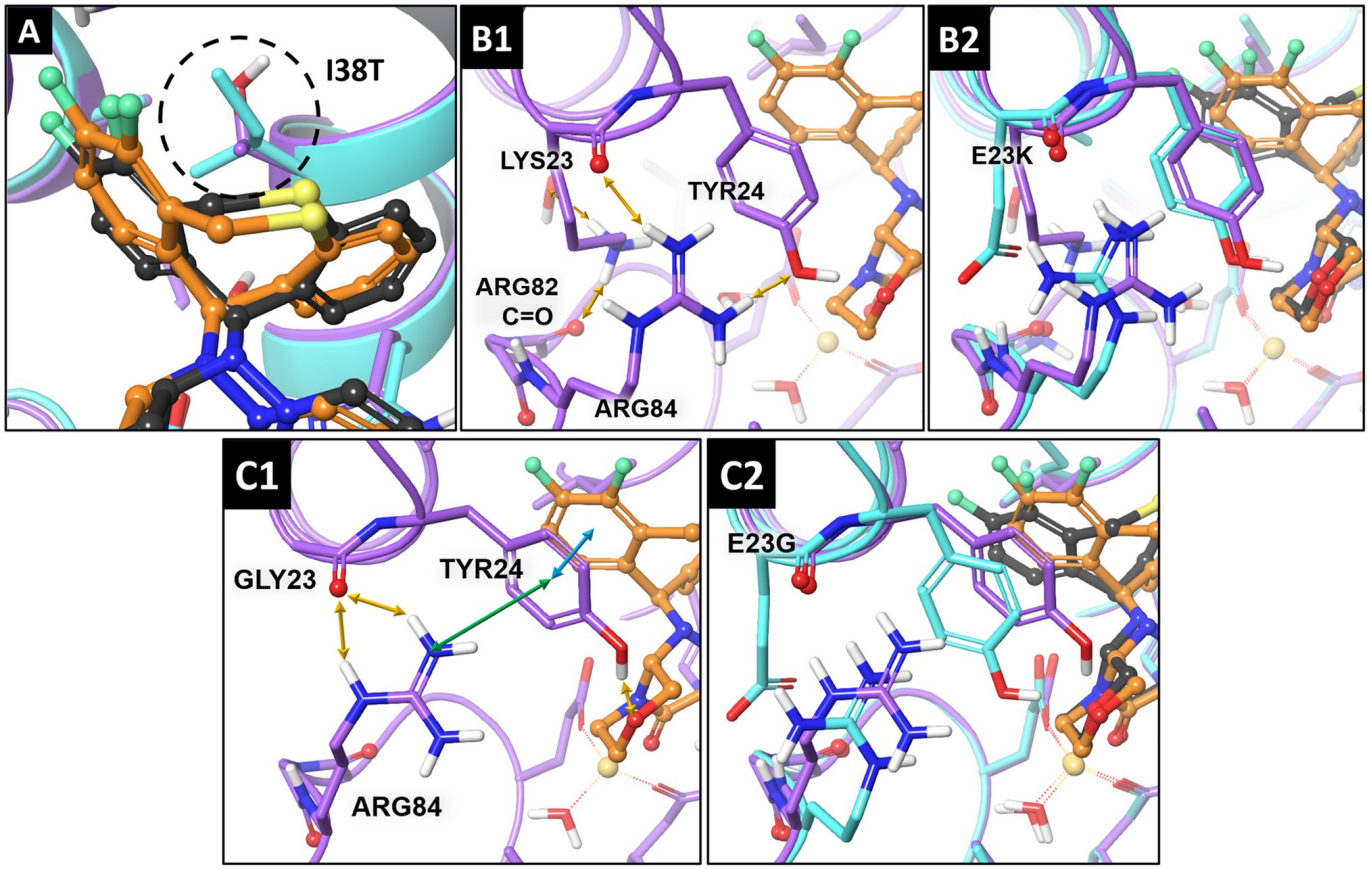
Modeling of the BXA-rPA_N_ binding interface of A(H1N1)pdm09 with E23G/K. **A)** The I38T substitution (dotted circle) leads to a significant decrease in van der Waals interactions between residue 38 and the hydrophobic sulfur-containing tricyclic system of BXA. **B1)** structural perturbations caused by E23K lead to conformational shifts of Arg84 and Tyr24 residues. **B2)** The aligned binding interfaces of BXA in WT and E23K PA_N_. **C1)** Structural perturbations caused by E23G lead to conformational shifts of Arg84 and Tyr24 residues. **C2)** The aligned binding interfaces of BXA in WT and E23G PA_N_. Light blue tubes: WT PA_N_; violet tubes: E23G/K-substituted PA_N_; dark-gray balls and sticks: the BXA pose in WT PA_N_; orange balls and sticks: the BXA pose in E23G/K-substituted PA_N_; gold arrows: hydrogen bonds; blue arrows: π-stacking interactions; green arrows: π-cation interactions.

For I38T PA_N_, the observed decrease in binding potency by a factor of 83 (based on plaque EC_50_s) (Table 1) can be explained from these structural perspectives, whereby the resulting interaction between the aromatic rings is now provided only by the Thr38 methyl group. Furthermore, the polarity of Thr38 exceeds that of Ile38 (Fig 7A). The remaining contacts are similar to those in the WT binding interface.

In contrast to WT PA_N_, in which the HB cluster between Glu23 and Arg84 contributes to BXA affinity, the E23G substitution causes a conformational shift within this segment (Fig 7C2). This results in the guanidine moiety of Arg84 interacting with Tyr24 through π-cationic stacking as a result of stabilization via two HBs formed with the Gly23 carbonyl atom. The HB between the -OH group of Tyr24 and morpholine oxygen, as well as the stacking interaction between aromatic rings of the ligand and Tyr24, are predicted for all the correct docking poses obtained (Fig 7C1). In E23K PA_N_, there is no stabilization between Arg84 and the basic nitrogen of lysine, which instead interacts with the carbonyl atom of Arg82 and with water molecules inside the site via HBs (Fig 7B1). The CH_2_ chain of Arg84 becomes flexible, provoking Tyr24 shifts (Fig 7B2) and weakening the stabilization with the carbonyl atom of Lys23. Furthermore, the HB between the Tyr24 -OH group and morpholine oxygen is missing from several docking poses for this substitution. Therefore, whereas both E23G and E23K can shift the Arg84–Tyr24 π-stacking stabilized complex, thereby reducing the interaction between Tyr24 and the BXA tricyclic moiety, E23K also disrupts Tyr24-OH interaction with the BXA morpholine oxygen of the metal-binding portion of the molecule; the combination may explain higher BXA EC_50_s for E23K vs. E23G.

**Fig 8:**
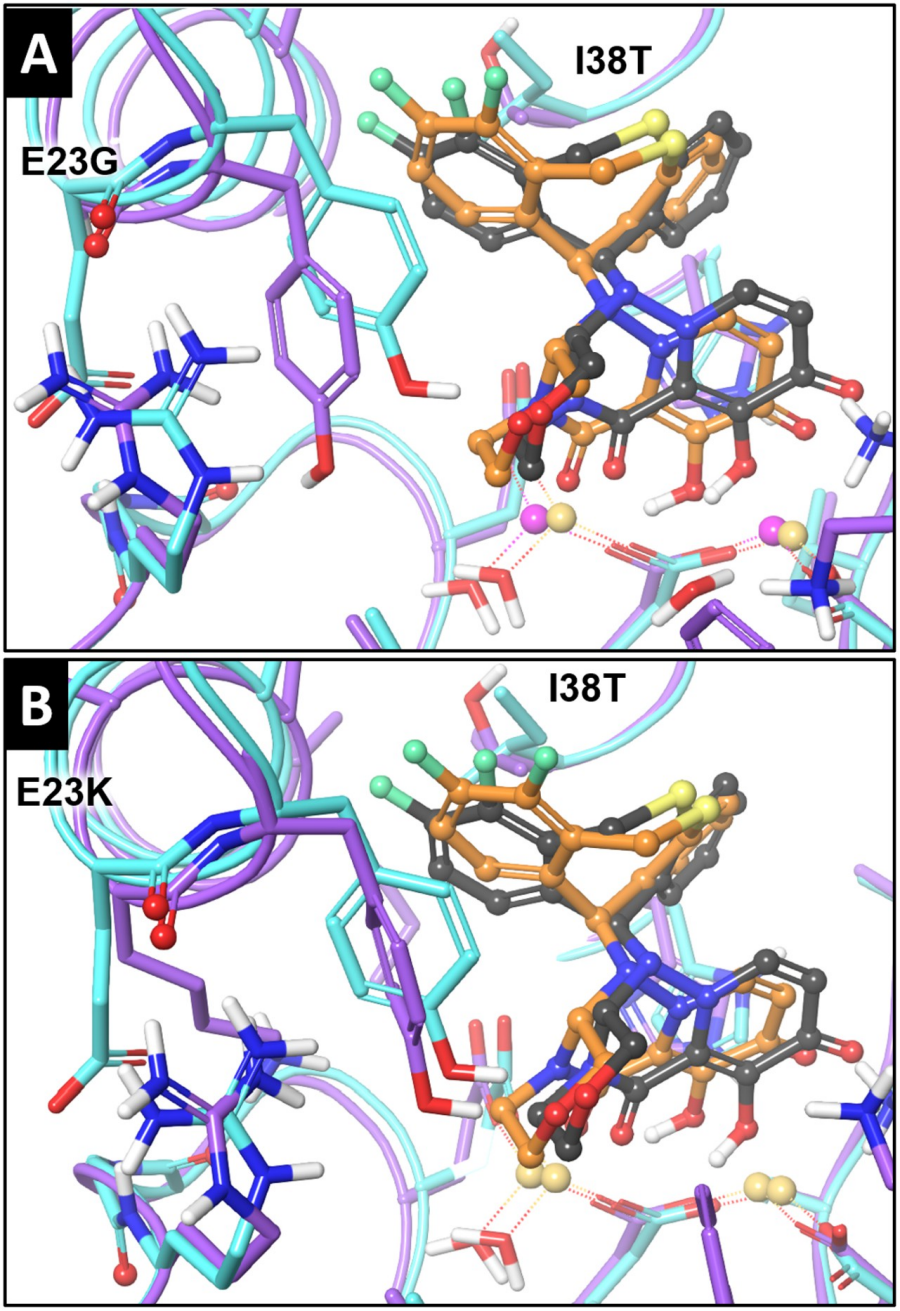
Modeling of the BXA-rPA_N_ binding interface of A(H1N1)pdm09 with E23G/K+I38T. **A)** The aligned binding interfaces of BXA in WT and E23G+I38T PA_N_. **B)** The aligned binding interfaces of BXA in WT and E23K+I38T PA_N_. Light blue tubes: WT PA_N_; violet tubes: E23G/K+I38T-substituted PA_N_; black balls and sticks: the BXA pose in WT PA_N_; orange balls and sticks: the BXA pose in E23G/K-substituted PA_N_.

A cumulative negative effect on BXA binding is observed with the dual substitutions E23G+I38T (Fig 8A) and E23K+I38T (Fig 8B). This correlates well with the docking scores obtained from the supramolecular interface misconfigurations, as compared to WT PA_N_ (S4 Table).

## Discussion

BXM was the first influenza antiviral to be widely approved in nearly two decades, supplementing the limited therapeutic options and offering a single-dose treatment regimen. Subsequent data revealed seasonal influenza viruses have a relatively low barrier to resistance, driven by PA 38 substitutions. BXA susceptibility and fitness dynamics of I38T/F/M viruses are well documented, but other substitutions, including E23G/K, are insufficiently characterized despite their inclusion in the WHO classifications of PA substitutions of concern for drug susceptibility [22]. We addressed this disparity by using recombinant E23G/K viruses with or without I38T substitution. Given the structural placement of E23 near the BXA binding site and reports of treatment-emergent E23X substitutions, we hypothesized that E23G/K would impart BXA resistance, but at a fitness cost, and that synergism with I38T would exacerbate both outcomes.

Although both E23G and E23K reduced virus susceptibility to BXA, the EC_50_s with E23K were greater than with E23G, following a trend reported within WHO influenza antiviral technical reports [2,22,29]. The opposite was true for in vitro replication, in which E23K viruses were generally more impaired in two separate cell lines, similar to the reported impairment of E23K A/Kanagawa/AC1929/2019 in MDCK cells [13]. Based on our modeling, we propose that E23G/K can disrupt BXA-PA_N_ binding through an indirect mechanism that involves destabilizing Arg84–Tyr24 conformations. Stabilization of the inherently flexible Tyr 24 [30,31] contributes to BXA binding, and we observed poses in which Tyr24 interacted with the fluorinated benzene of the upper tricyclic rings of BXA, as well as with the morpholine oxygen of the lower tricyclic rings. Whereas E23G is proposed to disrupt the contact in the upper rings, E23K disrupts both contacts in most of our models and may cause greater reductions in drug affinity. This predicted outcome was then experimentally demonstrated in our thermostability studies. When I38T is added, it further decreases drug affinity and increases EC_50_ fold-changes, both of which are more severe with E23K. The same E23–stabilized Tyr24 conformations that contribute to BXA binding also bind host RNA substrate, specifically through π-stacking with a nucleotide purine moiety and/or hydrogen bonding with a nucleotide ribose oxygen [30]. The more disrupted orientation caused by E23K may partly explain the more severe fitness defects caused by this substitution. However, the virus may sacrifice some replication potential to increase its propagation under drug pressure. A similar situation is observed with I38X substitutions, whereby I38M (particularly in influenza B) is fitter than I38T [19] but has much lower BXA resistance potential and is far less prevalent in clinical specimens. Finally, there are more reports of E23K isolation than of E23G isolation, providing additional evidence that E23K is the preferred substitution for the mechanisms discussed above.

The in vitro attenuation of E23G/K viruses may indicate a lower risk of successful replication or community spread. However, in vitro attenuations of I38T viruses did not predict a lack of transmission [4,19,23–25]. We previously observed airborne transmission of recombinant A(H1N1)pdm09 I38T virus to 3 of 3 exposed ferret contacts [19], while Imai et al. observed similar respiratory droplet transmission to 3 of 3 exposed ferret contacts with an I38T clinical isolate [23]. Similarly, in ferrets, we observed transmission of E23G/K to 3 of 3 exposed direct or airborne contacts, while only E23K+I38T virus was shed less by donors (vs. the single substitution) and exhibited incomplete airborne spread. The significance of one of three AC ferrets failing to shed virus is difficult to determine without resource-intensive large-scale ferret experiments. This study is also limited by exclusion of the parental WT in transmission studies. Therefore, additional analyses of E23X in ferrets, analysis of E23X in other subtypes [i.e. A(H3N2)], and potentially competitive mixtures of E23X with WT virus are important for ongoing evaluation of BXM. Nevertheless, our data clearly show that these viruses transmit via direct-contact and airborne routes, supporting the transmission hypotheses derived from multiple clinical reports. The E23K A/Kanagawa/AC1920/2019 isolate discussed previously was isolated from a primary school experiencing an influenza outbreak. This setting, along with the high BXM usage in Japan, led the authors to speculate that human-to-human transmission of the E23K genotype was possible [13]. In a separate study of BXM prophylaxis for household contacts of influenza-positive individuals, five E23K viruses were isolated, of which three were positive for the E23K sequence before BXM treatment of the contact patients began [9]. Currently, there are no clinical reports of E23G transmission. Therefore, influenza viruses with E23G/K substitutions are transmission competent in animal models and there is evidence of transmission events in humans. The risk posed by such viruses might be mitigated if reversion to WT drug-susceptible genotypes occurs during transmission. However, our data demonstrate that E23G/K genotypes remain stable in the absence of drug pressure.

No dual-substituted E23G/K+I38T treatment-emergent viruses have been described to date, but we have isolated this genotype using a BXA analogue [5]. Additionally, dual substitutions are described that confer resistance to adamantine drugs (M2 protein S31N+L26I or S31N+V27A) [32,33] and NAIs (NA H275Y+I223F) [34,35]. A similar phenomenon should, therefore, be anticipated for BXM. According to our data, PA E23G/K+I38T synergizes to not only decrease protein–drug target binding but also strongly increases EC_50_s. The virus that confers the highest EC_50_s (E23K+I38T) has some in vitro and in vivo fitness deficits. This may hinder productive spread of the virus to/from healthy individuals, but not necessarily to/from high-risk or immunocompromised patients, who are more likely to receive post-infection antiviral intervention and to exhibit prolonged virus shedding. A similar phenomenon was documented with a stem-cell transplant recipient who was treated with multiple NAIs and shed dual-resistant NA E119D+H275Y virus, resulting in viral rebound with a >5000-fold increase in NAI IC_50_s [36]. That E23K+I38T virus remains genetically stable further increases its potential to develop in immunocompromised patients, enter circulation, and spread among healthy individuals.

Future investigations of E23G/K substitutions will extend our understanding of their impact on BXM treatment. Our modeling has enabled us to propose mechanisms to explain the associated fitness deficits and resistance profiles. However, E23G/K PA_N_ co-crystallizations with BXA or RNA substrate are logical confirmatory studies which may compliment the in silico data to design novel or improved endonuclease inhibitors possessing little or no cross-reactive resistance to BXA. Further, such studies could also elucidate the structural impacts of E23G/K on other subtypes, helping to explain why E23G has not been observed to occur naturally in A(H3N2) viruses and why it failed to result in a >3-fold EC_50_ increase, the suggested threshold for BXA resistance [2,12,14]. Other substitutions at Glu23 may also influence BXM resistance, similar to the observations with I38X [18,37], but it will also be important to explore synergy between other BXA-associated PA substitutions like E23G/K/R, I38F/M/N/S, E199D/G, etc. Finally, the evidence for the contribution of E23X to influenza B virus infectivity is conflicting: E23K caused no EC_50_ increase in recombinant B/Maryland/1/1959 virus [6], but it imparted a modest 2.6-fold EC_50_ change accompanied by reduced polymerase activity and cell replication fitness in recombinant B/Phuket/3073/2013 virus [25]. These discrepancies may be due to the differing interactions of BXA with the influenza B PA_N_ [6], which has also strongly influenced the differences in BXA efficacy between I38X-substituted influenza A and B viruses [4,19,25].

In summary, we propose that, like I38T, E23G/K substitutions are important markers of BXM resistance that function to decrease drug binding by weakening key PA anchor points. E23G/K viruses are predominantly genetically stable, have WT-equivalent, or near-WT-equivalent airborne transmission potential, and synergize with I38T to markedly decrease inhibition by BXA.

## Materials and methods

### Ethics statement

All animal experiments within this study were approved by the St. Jude Children’s Research Hospital Animal Care and Use Committee in accordance with the Animal Welfare Act (USDA) and NIH Guide for the Care and Use of Laboratory Animals [38].

### Cells and compounds

Madin–Darby canine kidney cells (MDCKs) and human embryonic kidney cells (HEK293Ts) were obtained from American Type Culture Collection with distributor recommended media. BXA was purchased from MedChem Express.

### Viruses

Recombinant viruses were by rescued reverse-genetics method [39] using PA plasmids containing substitutions introduced with gene-specific primers and QuikChange Site-Directed Mutagenesis (Agilent). Viruses were propagated in MDCKs, 37°C, 72 h in infection medium (1×MEM, 1% BSA, 1 μg/mL l-tosylamido 2-phenylethyl chloromethyl ketone trypsin). Full-length PA gene was sequenced (Sanger, MiSeq) to confirm substitution(s).

### Recombinant endonuclease expression

Sequences encoding loop-deleted PA_N_ (residues 51–72 replaced by GGS linker) were expressed and purified as described [17].

### Minireplicon assay

Minireplicon luciferase reporter assays were conducted as described [17], with DMSO or BXA pre-incubation (1 hr) then polymerase protein or reporter plasmid (Meghan Shaw, Mt. Sinai School of Medicine) transfection. 24 h post transfection, ratios of luciferase (Luciferase Assay System [Promega]) to β-galactosidase were determined in cell lysates. Values were normalized to ratios from E23+I38-WT (100% polymerase activity) or I38T (100% BXA resistance) samples.

### Plaque reduction assay and plaque sizing

MDCKs (1×10^6^ cells/well) were inoculated (50–100 plaque-forming units [PFU]) for 1 h, washed, and overlaid with 0.45% immunodiffusion-grade agarose (MP Biomedical) in infection medium containing BXA (0 or 1 pM–1.5 μM). At 72 hpi, PFU/well were enumerated, and EC_50_s were determined with GraphPad Prism (log inhibitor vs. response logistic nonlinear regression). Separately, under no drug pressure, plaque monolayers (n = 6 wells) were prepared, and ≥87individual PFU/assay were sized by ImageJ (NIH).

### Replication kinetics

Replication curves were performed similar to plaque assays, but at MOI 0.001 or 0.01 and in 3 mL infection medium. Supernatants were sampled 2–96 hpi, titrated in MDCKs, and 50% tissue culture infectious doses (TCID_50_) values determined by Reed/Meunch method [40].

### PA substitution stability

MDCKs were plated as in plaque assays, and virus inoculated (MOI 0.01). When cytopathic effect (CPE) reached ≥75%, supernatants were harvested and blind-passaged (1:500 dilution virus to infection medium) 5 times in duplicate. Total RNA was isolated (RNA Easy, Qiagen) from P0 and P5, endonuclease segments were PCR amplified (Phusion, NEB), and PA genotypes determined with Illumina MiSeq with CLC Genomics Workbench v21 (Qiagen) with variant calling by method of Imai et al. [23] (variant frequency cut-offs of 5% with ≥1000 reads; 10% with <1000 reads).

### Ferret transmission experiments

Experimental groups were housed in individual isolators with negative airflow, with personal protective equipment changes and surface decontamination performed in between. Male ferrets 4–5 months (Triple F Farms) were seronegative for influenza A/B by hemagglutination inhibition (HI) assay [41] and SARS-CoV-2 seronegative by S and N-protein–ELISAs (Abclonal and SinoBiological, respectively) and rapid antigen tests (Beckman-Dickinson). Transmission experiments were performed as previously described [19]. Briefly, donors (n = 3/virus) were intranasally virus inoculated with 10^5^ TCID_50_ units of E23G/K virus, or the highest attainable inoculum from MDCK-grown E23G/K+I38T virus (1.8x10^4^ or 5.6x10^4^ TCID_50_ units), then housed 24 h later with DC (same cage) and AC (adjacent air-flow permissive cage that prevented contact). Biometrics and symptoms were recorded (≥20 min daily, S2 Table), and nasal washes were collected every 48 h by administering ketamine IM (25 mg/kg) and instilling 1 mL PBS intranasally, followed by virus titration. Seroconversion to homologous virus was determined by HI assay. AUC shedding was calculated with GraphPad Prism. Each virus-positive nasal wash was subjected to PA genotype analysis as described previously.

### Thermal stability assays

Binding and thermal stabilization of BXA–rPA_N_ was performed as described previously [6] in modified thermofluor assays. rPA_N_ (2 μM) was incubated with vehicle (DMSO) or BXA (37 μM), along with 4× SYPRO Orange (Sigma) in assay buffer (100 mM NaCl, 20 mM Tris-HCL, pH 8.3, supplemented before assays with 10 mM BME, 5 mM MnCl_2_). Samples were heated 1°C/min from 30 to 90°C, and emitted SYPRO signal was read at each step. GraphPad Prism was used to plot values and derive T_m_s and ΔT_m_s (vehicle vs. BXA treated curves).

### In silico modeling

Maestro software (Schrödinger) was used to perform preprocessing and energy minimization for ligand–protein complexes (BXA-A/California/04/2009 PA_N_) (pdb codes: 6FS6-WT, 5VPX-I38T). BXA was properly placed within the native binding site before minimization, with stepwise visual inspection and consideration of reported binding modes. Conservative H_2_0 were retained. Point mutations were introduced (Maestro; 6FS6-E23K/G, 5VPX- E23K/G+I38T). Complexes were preprocessed and minimized in Maestro according to standard protocol. Docking studies used induced-fit mode [28] with Mn^2+^ as a crucial binding point. Four scores were obtained: Docking_score, Glide_emodel, D_score ([Docking_score]×[Glide_emodel]), and Max._score. Several predicted binding modes (poses) were output; poses distant from the original BXA binding mode were excluded. Consequently, each score was calculated as the mean and median values. Docking scores were converted to + scale by ×(−1); higher scores corresponded to high activity and vice versa. Resulting scores and QSAR equations based on EC_50_ values are provided (S4 Table). For consistency, Figs 5–8 are optimal (best-scoring) poses.

### Statistical analysis

Data were analyzed using unpaired *t*-tests, two-way ANOVA, and univariant log-rank analysis (survival curves) in GraphPad Prism_v9. Replicates, group comparisons, and *P* values are listed in each figure legend.

## Supporting information

S1 Table PA E23 amino acid variance among human influenza A viruses. (DOCX)

S2 Table Pathogenicity, transmission, and seroconversion of ferrets inoculated with influenza A(H1N1)pdm09 viruses with PA E23G/K and E23G/K+I38T. (DOCX)

S3 Table Significance (P values) between thermostability assay ΔTms by 1-way ANOVA. (DOCX)

S4 Table In silico induced-fit docking scores for BXA and influenza A virus PA substitutions and QSAR-modelling results based on correlation between scores and antiviral activities of BXA. (DOCX)

S1 File Data sets. (XLSX)

## References

[1] Abed Y, Boivin G. A Review of Clinical Influenza A and B Infections With Reduced Susceptibility to Both Oseltamivir and Zanamivir. Open Forum Infect Di. 2017;4(3). doi: 10.1093/ofid/ofx105 28852674 PMC5569976

[2] Ison MG, Hayden FG, Hay AJ, Gubareva LV, Govorkova EA, Takashita E, et al. Influenza polymerase inhibitor resistance: Assessment of the current state of the art—A report of the isirv Antiviral group. Antiviral Res. 2021;194:105158. doi: 10.1016/j.antiviral.2021.105158 34363859 PMC9012257

[3] Bai Y, Jones JC, Wong SS, Zanin M. Antivirals Targeting the Surface Glycoproteins of Influenza Virus: Mechanisms of Action and Resistance. Viruses. 2021;13(4). doi: 10.3390/v13040624 33917376 PMC8067422

[4] Abed Y, Saim-Mamoun A, Boivin G. Fitness of influenza A and B viruses with reduced susceptibility to baloxavir: A mini-review. Rev Med Virol. 2021;31(3):e2175. doi: 10.1002/rmv.2175 32975358

[5] Jones JCa, Kumar G, Barman S, Najera I, White SW, Webby RJ, et al. Identification of the I38T PA Substitution as a Resistance Marker for Next-Generation Influenza Virus Endonuclease Inhibitors. mBio. 2018;9(2). 10.1128/mBio.00430-18 PMC5915737 29691337

[6] Omoto S, Speranzini V, Hashimoto T, Noshi T, Yamaguchi H, Kawai M, et al. Characterization of influenza virus variants induced by treatment with the endonuclease inhibitor baloxavir marboxil. Sci Rep. 2018;8(1):9633. doi: 10.1038/s41598-018-27890-4 29941893 PMC6018108

[7] Hayden FG, Sugaya N, Hirotsu N, Lee N, de Jong MD, Hurt AC, et al. Baloxavir Marboxil for Uncomplicated Influenza in Adults and Adolescents. N Engl J Med. 2018;379(10):913–23. doi: 10.1056/NEJMoa1716197 30184455

[8] Hirotsu N, Sakaguchi H, Sato C, Ishibashi T, Baba K, Omoto S, et al. Baloxavir Marboxil in Japanese Pediatric Patients With Influenza: Safety and Clinical and Virologic Outcomes. Clin Infect Dis. 2020;71(4):971–81. doi: 10.1093/cid/ciz908 31538644 PMC7428393

[9] Ikematsu H, Hayden FG, Kawaguchi K, Kinoshita M, de Jong MD, Lee N, et al. Baloxavir Marboxil for Prophylaxis against Influenza in Household Contacts. N Engl J Med. 2020;383(4):309–20. doi: 10.1056/NEJMoa1915341 32640124

[10] Takashita Ea, Kawakami C, Ogawa R, Morita H, Fujisaki S, Shirakura M, et al. Influenza A(H3N2) virus exhibiting reduced susceptibility to baloxavir due to a polymerase acidic subunit I38T substitution detected from a hospitalised child without prior baloxavir treatment, Japan, January 2019. Euro Surveill. 2019;24(12). 10.2807/1560-7917.ES.2019.24.12.1900170 PMC6440584 30914078

[11] Takashita Eb, Ichikawa M, Morita H, Ogawa R, Fujisaki S, Shirakura M, et al. Human-to-Human Transmission of Influenza A(H3N2) Virus with Reduced Susceptibility to Baloxavir, Japan, February 2019. Emerg Infect Dis. 2019;25(11):2108–11. doi: 10.3201/eid2511.190757 31436527 PMC6810216

[12] Ince WL, Smith FB, O’Rear JJ, Thomson M. Treatment-Emergent Influenza Virus Polymerase Acidic Substitutions Independent of Those at I38 Associated With Reduced Baloxavir Susceptibility and Virus Rebound in Trials of Baloxavir Marboxil. J Infect Dis. 2020;222(6):957–61. doi: 10.1093/infdis/jiaa164 32253432

[13] Takashita Ed, Abe T, Morita H, Nagata S, Fujisaki S, Miura H, et al. Influenza A(H1N1)pdm09 virus exhibiting reduced susceptibility to baloxavir due to a PA E23K substitution detected from a child without baloxavir treatment. Antiviral Res. 2020;180:104828. doi: 10.1016/j.antiviral.2020.104828 32574689

[14] Gubareva LV, Mishin VP, Patel MC, Chesnokov A, Nguyen HT, De La Cruz J, et al. Assessing baloxavir susceptibility of influenza viruses circulating in the United States during the 2016/17 and 2017/18 seasons. Euro Surveill. 2019;24(3). doi: 10.2807/1560-7917.ES.2019.24.3.1800666 30670144 PMC6344838

[15] Takashita Ec, Daniels RS, Fujisaki S, Gregory V, Gubareva LV, Huang W, et al. Global update on the susceptibilities of human influenza viruses to neuraminidase inhibitors and the cap-dependent endonuclease inhibitor baloxavir, 2017–2018. Antiviral Res. 2020;175:104718. doi: 10.1016/j.antiviral.2020.104718 32004620

[16] Food and Drug Administration US. XOFLUZA^®^ (baloxavir marboxil) tablets, for oral use. In: Services USDoHaH, editor. 2019.

[17] Song MS, Kumar G, Shadrick WR, Zhou W, Jeevan T, Li Z, et al. Identification and characterization of influenza variants resistant to a viral endonuclease inhibitor. Proc Natl Acad Sci U S A. 2016;113(13):3669–74. doi: 10.1073/pnas.1519772113 26976575 PMC4822642

[18] Jones JC, Pascua PNQ, Harrington WN, Webby RJ, Govorkova EA. Multiple polymerase acidic (PA) I38X substitutions in influenza A(H1N1)pdm09 virus permit polymerase activity and cause reduced baloxavir inhibition. J Antimicrob Chemother. 2021;76(4):957–60. doi: 10.1093/jac/dkaa527 33351916 PMC7953318

[19] Jones JC, Pascua PNQ, Fabrizio TP, Marathe BM, Seiler P, Barman S, et al. Influenza A and B viruses with reduced baloxavir susceptibility display attenuated in vitro fitness but retain ferret transmissibility. Proc Natl Acad Sci U S A. 2020;117(15):8593–601. doi: 10.1073/pnas.1916825117 32217734 PMC7165484

[20] Hickerson BTA, S.E.; Barman, S.; Miller, L.; Lugovtsev, V.Y.; Webby, R.J.: Ince, W.; Donnelly, R.P.; Ilyushina, N.A. https://journals.asm.org/doi/epub/10.1128/aac.00009-22. Antimicrob Agents Chemother. 2022;In press. 10.1128/aac.00009-22 PMC9017380 35262375

[21] Takashita E, Abe T, Morita H, Nagata S, Fujisaki S, Miura H, et al. Influenza A(H1N1)pdm09 virus exhibiting reduced susceptibility to baloxavir due to a PA E23K substitution detected from a child without baloxavir treatment. Antiviral Res. 2020a;180:104828. doi: 10.1016/j.antiviral.2020.104828 32574689

[22] World Health Organization. Summary of polymerase acidic (PA) protein amino acid substitutions analysed for their effects on baloxavir susceptibility. In: WEP GIP, editor. 2021.

[23] Imai M, Yamashita M, Sakai-Tagawa Y, Iwatsuki-Horimoto K, Kiso M, Murakami J, et al. Influenza A variants with reduced susceptibility to baloxavir isolated from Japanese patients are fit and transmit through respiratory droplets. Nat Microbiol. 2020;5(1):27–33. doi: 10.1038/s41564-019-0609-0 31768027 PMC13014278

[24] Checkmahomed L, M’Hamdi Z, Carbonneau J, Venable MC, Baz M, Abed Y, et al. Impact of the Baloxavir-Resistant Polymerase Acid I38T Substitution on the Fitness of Contemporary Influenza A(H1N1)pdm09 and A(H3N2) Strains. J Infect Dis. 2020;221(1):63–70. doi: 10.1093/infdis/jiz418 31419295 PMC6910874

[25] Abed Y, Fage C, Checkmahomed L, Venable MC, Boivin G. Characterization of contemporary influenza B recombinant viruses harboring mutations of reduced susceptibility to baloxavir marboxil, in vitro and in mice. Antiviral Res. 2020;179:104807. doi: 10.1016/j.antiviral.2020.104807 32343991

[26] Pascua PNQ, Jones JC, Marathe BM, Seiler P, Caufield WV, Freeman BB 3rd, et al. Baloxavir Treatment Delays Influenza B Virus Transmission in Ferrets and Results in Limited Generation of Drug-Resistant Variants. Antimicrob Agents Chemother. 2021;65(11):e0113721. doi: 10.1128/AAC.01137-21 34424039 PMC8522732

[27] Belser JA, Eckert AM, Huynh T, Gary JM, Ritter JM, Tumpey TM, et al. A Guide for the Use of the Ferret Model for Influenza Virus Infection. Am J Pathol. 2020;190(1):11–24. doi: 10.1016/j.ajpath.2019.09.017 31654637 PMC8264465

[28] Miller EB, Murphy RB, Sindhikara D, Borrelli KW, Grisewood MJ, Ranalli F, et al. Reliable and Accurate Solution to the Induced Fit Docking Problem for Protein-Ligand Binding. J Chem Theory Comput. 2021;17(4):2630–9. doi: 10.1021/acs.jctc.1c00136 33779166

[29] Govorkova EA, Takashita E, Daniels RS, Fujisaki S, Presser LD, Patel MC, et al. Global update on the susceptibilities of human influenza viruses to neuraminidase inhibitors and the cap-dependent endonuclease inhibitor baloxavir, 2018–2020. Antiviral Res. 2022:105281. doi: 10.1016/j.antiviral.2022.105281 35292289 PMC9254721

[30] Kumar G, Cuypers M, Webby RR, Webb TR, White SW. Structural insights into the substrate specificity of the endonuclease activity of the influenza virus cap-snatching mechanism. Nucleic Acids Res. 2021;49(3):1609–18. doi: 10.1093/nar/gkaa1294 33469660 PMC7897473

[31] Stevaert A, Naesens L. The Influenza Virus Polymerase Complex: An Update on Its Structure, Functions, and Significance for Antiviral Drug Design. Med Res Rev. 2016;36(6):1127–73. doi: 10.1002/med.21401 27569399 PMC5108440

[32] Abed Y, Goyette N, Boivin G. Generation and characterization of recombinant influenza A (H1N1) viruses harboring amantadine resistance mutations. Antimicrob Agents Chemother. 2005;49(2):556–9. doi: 10.1128/AAC.49.2.556-559.2005 15673732 PMC547263

[33] Musharrafieh R, Ma C, Wang J. Discovery of M2 channel blockers targeting the drug-resistant double mutants M2-S31N/L26I and M2-S31N/V27A from the influenza A viruses. Eur J Pharm Sci. 2020;141:105124. doi: 10.1016/j.ejps.2019.105124 31669761 PMC6951800

[34] Tan H, Wei K, Bao J, Zhou X. In silico study on multidrug resistance conferred by I223R/H275Y double mutant neuraminidase. Mol Biosyst. 2013;9(11):2764–74. doi: 10.1039/c3mb70253g 24056678

[35] van der Vries E, Collins PJ, Vachieri SG, Xiong X, Liu J, Walker PA, et al. H1N1 2009 pandemic influenza virus: resistance of the I223R neuraminidase mutant explained by kinetic and structural analysis. PLoS Pathog. 2012;8(9):e1002914. doi: 10.1371/journal.ppat.1002914 23028314 PMC3447749

[36] L’Huillier AG, Abed Y, Petty TJ, Cordey S, Thomas Y, Bouhy X, et al. E119D Neuraminidase Mutation Conferring Pan-Resistance to Neuraminidase Inhibitors in an A(H1N1)pdm09 Isolate From a Stem-Cell Transplant Recipient. J Infect Dis. 2015;212(11):1726–34. doi: 10.1093/infdis/jiv288 25985905 PMC4633758

[37] Hashimoto T, Baba K, Inoue K, Okane M, Hata S, Shishido T, et al. Comprehensive assessment of amino acid substitutions in the trimeric RNA polymerase complex of influenza A virus detected in clinical trials of baloxavir marboxil. Influenza Other Respir Viruses. 2021;15(3):389–95. doi: 10.1111/irv.12821 33099886 PMC8051730

[38] National Research Council. Guide for the Care and Use of Laboratory Animals. 8 ed. Washington, DC: National Academics Press; 2011.

[39] Hoffmann E, Krauss S, Perez D, Webby R, Webster RG. Eight-plasmid system for rapid generation of influenza virus vaccines. Vaccine. 2002;20(25–26):3165–70. doi: 10.1016/s0264-410x(02)00268-2 12163268

[40] Reed LJ M H. A simple method of estimating fifty percent endpoints. American Journal of Epidemiology. 1938;27(3):493–7.

[41] Kim JK, Kayali G, Walker D, Forrest HL, Ellebedy AH, Griffin YS, et al. Puzzling inefficiency of H5N1 influenza vaccines in Egyptian poultry. Proc Natl Acad Sci U S A. 2010;107(24):11044–9. doi: 10.1073/pnas.1006419107 20534457 PMC2890765

