# Supplementary material for: Influenza A virus polymerase acidic protein E23G/K substitutions weaken key baloxavir drug-binding contacts with minimal impact on replication and transmission
Source: PLoS Pathog. 2022 Jul 13;18(7):e1010698. doi: 10.1371/journal.ppat.1010698 (PMC9312377; doi:10.1371/journal.ppat.1010698)
Supplement: S1 Table — (DOCX) [file ppat.1010698.s001.docx]

**Supplemental Table 1. PA E23 amino acid variance among human influenza A viruses**.

| **Subtype** | **No. PA protein sequences available^a^** | **No. isolates with PA 23 identity:** | | | |
| --- | --- | --- | --- | --- | --- |
|  |  | **E** | **K** | **G** | **Other** |
| H1N1 | 15801 | 15796 | 1 | 4 | 0 |
| H2N2 | 111 | 111 | 0 | 0 | 0 |
| H3N2 | 21423 | 21423 | 0 | 0 | 0 |
| H5N1 | 275 | 275 | 0 | 0 | 0 |
| H7N9 | 176 | 176 | 0 | 0 | 0 |
| H9N2 | 33 | 33 | 33 | 0 | 0 |
| ^a^ Influenza Research Database, accessed 9.17.2021 (https://www.fludb.org/brc/home.spg?decorator=influenza) | | | | | |
| ^b^ Protein sequences were obtained from pre-computed IRD data sets and therefore, may exclude individual E23X substitutions cited within this manuscript. | | | | | |
