# Supplementary material for: Influenza A virus polymerase acidic protein E23G/K substitutions weaken key baloxavir drug-binding contacts with minimal impact on replication and transmission
Source: PLoS Pathog. 2022 Jul 13;18(7):e1010698. doi: 10.1371/journal.ppat.1010698 (PMC9312377; doi:10.1371/journal.ppat.1010698)
Supplement: S2 Table — (DOCX) [file ppat.1010698.s002.docx]

**Supplemental Table 2. Pathogenicity, transmission, and seroconversion of ferrets inoculated with influenza A(H1N1)pdm09 viruses with PA E23G/K and E23G/K+I38T.**

| Virus^a^ | Contact status^b^ | Clinical events^c^ | Respiratory events^d^ | Onset of shedding^e^ | Range of shedding^f^ | Shedding/ transmission^g^ | Seroconversion^h^ | HAI titer^i^ |
| --- | --- | --- | --- | --- | --- | --- | --- | --- |
| E23K | Donors | 4 | 14 | 2 (3/3) | 2 to 4 | 3/3 | 3/3 | 320 to 640 |
|  | DC | 1 | 9 | 4 (3/3) | 4 to 8 | 3/3 | 3/3 | 160 to 320 |
|  | AC | 9 | 13 | 2 (1/3) | 2 to 10 | 3/3 | 3/3 | 320 |
| E23K+I38T | Donors | 2 | 10 | 2 (3/3) | 2 | 3/3 | 3/3 | 160 to 640 |
|  | DC | 0 | 9 | 4 (3/3) | 4 to 8 | 3/3 | 3/3 | 160 to 320 |
|  | AC | 0 | 3 | 8 (1/3) | 8 to 12 | 2/3 | 3/3 | 160 to 320 |
| E23G | Donors | 4 | 7 | 2 (3/3) | 2 to 4 | 3/3 | 3/3 | 160 to 640 |
|  | DC | 1 | 9 | 4 (3/3) | 4 to 6 | 3/3 | 3/3 | 160 to 640 |
|  | AC | 2 | 12 | 4 (1/3) | 4 to 10 | 3/3 | 3/3 | 160 to 320 |
| E23G+I38T | Donors | 8 | 11 | 2 (3/3) | 2 to 4 | 3/3 | 3/3 | 160 to 2560 |
|  | DC | 0 | 5 | 4 (3/3) | 4 to 6 | 3/3 | 3/3 | 80 to 320 |
|  | AC | 6 | 14 | 4 (2/3) | 4 to 10 | 3/3 | 3/3 | 80 to 320 |
| ^a^ rgA/California/04/2009 (H1N1)pdm09 with the indicated PA endonuclease domain substitutions(s). | | | | | | | | |
| ^b^ Donors were directly inoculated and paired with direct (DC) or airborne contacts (AC) as described in the Materials and Methods section. | | | | | | | | |
| ^c^ Total instances of lethargy, fever, and/or anorexia among all animals within the virus group. | | | | | | | | |
| ^d^ Total instances of sneezing, coughing, and/or congested breathing among all animals within the virus group. | | | | | | | | |
| ^e^ Days post donor inoculation with a positive nasal wash in at least one ferret; values in parentheses indicate the number of ferrets meeting this criterion out of the total number in the indicated contact status. | | | | | | | | |
| ^f^ Days post-donor inoculation | | | | | | | | |
| ^g^ Number of animals shedding virus at one or more time points out of the total number in the indicated contact status | | | | | | | | |
| ^h^ Number of ferrets exhibiting serum neutralizing titers of ≥ 1:40 (reciprocal endpoint dilution titer) to homologous virus out of the total group. | | | | | | | | |
| ^i^ Range of hemagglutination inhibition titers for animals in the indicated contact status. | | | | | | | | |
