# Supplementary material for: Influenza A virus polymerase acidic protein E23G/K substitutions weaken key baloxavir drug-binding contacts with minimal impact on replication and transmission
Source: PLoS Pathog. 2022 Jul 13;18(7):e1010698. doi: 10.1371/journal.ppat.1010698 (PMC9312377; doi:10.1371/journal.ppat.1010698)
Supplement: S3 Table — (DOCX) [file ppat.1010698.s003.docx]

**Supplemental Table 3. Significance (*P* values) between thermostability assay ΔT_m_s by 1-way ANOVA^a^**.

| **PA_N_ origin^b^** | **PA_N_ Substitution** | **I38** | **I38T** | **E23G** | **E23G+I38T** | **E23K** | **E23K+I38T** |
| --- | --- | --- | --- | --- | --- | --- | --- |
| A(H1N1)pdm09 | **I38** | - | *P* < 0.0001 | *P* < 0.0001 | *P* < 0.0001 | *P* < 0.0001 | *P* < 0.0001 |
|  | **I38T** | *P* < 0.0001 | - | *P* < 0.0001 | *P* < 0.0001 | *P* < 0.0001 | *P* < 0.001 |
|  | **E23G** | *P* < 0.0001 | *P* < 0.0001 | - | *P* < 0.0001 | *P* < 0.001^c^ | *P* < 0.0001 |
|  | **E23G+I38T** | *P* < 0.0001 | *P* < 0.0001 | *P* < 0.0001 | - | *P* < 0.0001 | NS^c^ |
|  | **E23K** | *P* < 0.0001 | *P* < 0.0001 | *P* < 0.001c | *P* < 0.0001 | - | *P* < 0.0001 |
|  | **E23K+I38T** | *P* < 0.0001 | *P* < 0.001 | *P* < 0.0001 | NS^c^ | *P* < 0.0001 | - |
| A(H3N2) | **I38** | - | *P* < 0.0001 | *P* < 0.0001 | *P* < 0.0001 | *P* < 0.01 | *P* < 0.001 |
|  | **I38T** | *P* < 0.0001 | - | *P* < 0.0001 | *P* < 0.001 | *P* < 0.0001^c^ | NS |
|  | **E23G** | *P* < 0.0001 | *P* < 0.0001 | - | *P* < 0.0001 | *P* < 0.01^c^ | *P* < 0.001 |
|  | **E23G+I38T** | *P* < 0.0001 | *P* < 0.001 | *P* < 0.0001 | - | *P* < 0.0001 | NS^c^ |
|  | **E23K** | *P* < 0.01 | *P* < 0.0001^c^ | *P* < 0.01^c^ | *P* < 0.0001 | - | *P* < 0.001 |
|  | **E23K+I38T** | *P* < 0.0001 | NS | *P* < 0.001 | NS^c^ | *P* < 0.001 | - |
| ^a^1-way ANOVA conducted in GraphPad Prism, using Geisser-Greenhouse correction, and matching experimental samples for each replicate assay. | | | | | | | |
| ^b^Recombinant influenza A, N-terminal endonuclease domain. | | | | | | | |
| ^c^1-way ANOVA analysis assuming no matching or pairing between each replicate assay yielded non-significance [*P* > 0.05]. | | | | | | | |
