# Supplementary material for: Influenza A virus polymerase acidic protein E23G/K substitutions weaken key baloxavir drug-binding contacts with minimal impact on replication and transmission
Source: PLoS Pathog. 2022 Jul 13;18(7):e1010698. doi: 10.1371/journal.ppat.1010698 (PMC9312377; doi:10.1371/journal.ppat.1010698)
Supplement: S4 Table — (DOCX) [file ppat.1010698.s004.docx]

**Supplemental Table 4. In silico induced-fit docking scores for BXA and influenza A virus PA substitutions and QSAR-modelling results based on correlation between scores and antiviral activities of BXA.**

| **Average values^a^** | | | | |
| --- | --- | --- | --- | --- |
| PA_N_ isoform^b^ | EC_50_^c^ | Docking score^d^ | Glide eModel | D score^e^ |
| WT | 0.22 | 7.06 | 81.90 | 588.45 |
| I38T | 18.27 | 5.95 | 71.79 | 432.18 |
| E23K | 2.8 | 6.80 | 74.91 | 524.17 |
| E23K + I38T | 48.3 | 5.85 | 68.32 | 407.92 |
| E23G | 1.63 | 7.12 | 78.41 | 558.33 |
| E23G + I38T | 30.42 | 5.98 | 70.95 | 424.39 |
| QSAR equation | | y = -0.82x + 7.23 | y = -7.24x + 82.68 | y = -114.92x + 602.7 |
| R^2^ | | R² = 0.88 | R² = 0.85 | R² = 0.91 |
| **MAX values^f^** | | | | |
| PA_N_ isoform | EC_50_ | Docking score | Glide eModel | D score |
| WT | 0.22 | 8.23 | 92.31 | 760.02 |
| I38T | 18.27 | 7.10 | 78.50 | 557.32 |
| E23K | 2.8 | 8.07 | 86.41 | 697.49 |
| E23K + I38T | 48.3 | 7.37 | 79.70 | 586.77 |
| E23G | 1.63 | 7.91 | 84.00 | 664.30 |
| E23G + I38T | 30.42 | 6.08 | 72.03 | 437.80 |
| QSAR equation | | y = -0.69x + 7.97 | y = -6.99x + 87.32 | y = -109.41x + 698.08 |
| R^2^ | | R² = 0.60 | R² = 0.80 | R² = 0.73 |
| * Scores calculated from the induced-fit docking poses that are similar to the intrinsic baloxavir binding mode. | | | | |
| ^a^ Average score for the 5–7 top docking poses that are similar to the intrinsic baloxavir binding mode for a specific variant. | | | | |
| ^b^ Structures were obtained from existing PDBs (WT: 6FS6 and 7KOW; I38T: 5VPX and 6SF7), or E23X substitutions were introduced (6SF6: E23G/K; 5VPX: E23G/K+I38T). | | | | |
| ^c^ 50% effective concentrations of BXA for H1N1 (Table 1). | | | | |
| ^d^ Free energy of binding scores corresponding to docking poses similar to the intrinsic BXA binding mode were converted to positive values ×(−1), where a higher score = greater activity/interaction potential. | | | | |
| ^e^ [Docking_score] × [Glide_emodel] | | | | |
| ^f^ The score for the best-scoring docking pose among the 5–7 top docking poses that are similar to the intrinsic baloxavir binding mode for a specific variant. | | | | |
